# Genetic analysis of stress hormone levels in hair of healthy nursery pigs and their relationships with backtest responses

**DOI:** 10.1093/genetics/iyaf092

**Published:** 2025-05-14

**Authors:** Fazhir Kayondo, Hayder Al-Shanoon, Yolande M Seddon, Dylan Carette, Carmen Cole, David M Janz, Frederic Fortin, John C S Harding, Michael K Dyck, Graham S Plastow, PigGen Canada, Jack C M Dekkers

**Affiliations:** Department of Animal Science, Iowa State University, Ames, IA 50011, USA; Western College of Veterinary Medicine, University of Saskatchewan, Saskatoon, SK S7N 5B4, Canada; Western College of Veterinary Medicine, University of Saskatchewan, Saskatoon, SK S7N 5B4, Canada; Western College of Veterinary Medicine, University of Saskatchewan, Saskatoon, SK S7N 5B4, Canada; Western College of Veterinary Medicine, University of Saskatchewan, Saskatoon, SK S7N 5B4, Canada; Western College of Veterinary Medicine, University of Saskatchewan, Saskatoon, SK S7N 5B4, Canada; Centre de Devéloppement du Porc du Québec Inc., Québec City, QC G1V 4M6, Canada; Western College of Veterinary Medicine, University of Saskatchewan, Saskatoon, SK S7N 5B4, Canada; Department of Agricultural, Food and Nutritional Science, University of Alberta, Edmonton, AB T6G 2R3, Canada; Department of Agricultural, Food and Nutritional Science, University of Alberta, Edmonton, AB T6G 2R3, Canada; PigGen Canada Research Consortium, Guelph, ON N1H4G8, Canada; Department of Animal Science, Iowa State University, Ames, IA 50011, USA

**Keywords:** swine, stress hormones, hair, backtest, genetics, glucocorticoid receptor

## Abstract

This study explored the genetics of the levels of stress hormones (cortisol, cortisone, DHEA, and DHEA-S) in hair of 863 clinically healthy Yorkshire × Landrace male pigs at ∼40 days of age and evaluated their potential as biomarkers of innate stress response by estimating genetic correlations with responses to a 30 s backtest performed at ∼27 days of age. Backtest responses included the number and intensity of vocalizations (VN and VI) and struggles (SN and SI). With pigs genotyped using a 50 K single nucleotide polymorphism (SNP) panel that was imputed to 650 K SNPs, heritability estimates for the levels of cortisol, cortisone, DHEA, and DHEA-S were 0.33, 0.04, 0, and 0.31, respectively, while those for backtest responses ranged from 0.26 to 0.57. Litter effects accounted for 9 to 16% of the phenotypic variance for stress hormone levels and none for backtest responses. Genetic correlation estimates among stress hormone levels were strongest between cortisol and cortisone (0.99 ± 0.12), while those among backtest responses ranged from 0.60 to 0.99. Cortisol was estimated to have moderate genetic correlations with VN (0.24 ± 0.19) and VI (0.50 ± 0.24) but not with SN and SI. Genome-wide association studies identified a major quantitative trait locus (QTL) for hair cortisol levels near the glucocorticoid receptor gene (*NR3C1*) that explained 45.3% of the genetic variance and that may be different than a causative mutation that was previously identified in this gene for cortisol levels in porcine blood. An extra copy of the minor allele (frequency = 9%) at the lead SNP for this QTL, rs341258564 originated from both parental breeds and reduced levels of cortisol by 30 ± 6% and of cortisone by 17 ± 4%, and increased VN by 5 ± 2%. Additional QTL with smaller effects (1.0 to 11.1% of genetic variance) were identified for DHEA-S, cortisol/DHEA-S, cortisone/DHEA-S, VI, and VN. Ranked gene set enrichment analyses of 0.25 Mb windows based on genetic variance explained showed that windows associated with glucocorticoid levels were enriched for biological terms related to energy production and suppression of inflammation. In contrast, those associated with DHEA-S were enriched for biological processes related to immunity activation and gene transcriptional and post-transcriptional regulation. These findings establish the genetic basis of stress response in young and clinically healthy pigs, identify the genomic location of a major QTL for hair cortisol levels, and show that cortisol levels in hair of young and healthy pigs are potential genetic biomarkers for the innate coping response style of pigs to noninfectious stressors. These results open avenues that can facilitate selection of pigs that cope better with noninfectious stressors.

## Teaser

Young pigs experience multiple non-infectious stressors, producing stress hormones that are captured in hair. Kayondo et al. determined the genetic basis of the levels of stress hormones in hair of young, clinically healthy pigs. The authors found that some are heritable, and that the hair cortisol level is a potential genetic biomarker of a pig’s coping style to non-infectious stress. They also identified a major gene for cortisol levels in hair. Results can facilitate the selection of pigs that cope better with non-infectious stressors.

## Introduction

Pork is one of the most popular animal proteins and is an efficient meat to produce (Gerbens-Leenes *et al.* 2013). It is the third most-consumed meat in the United States and currently the second most-consumed globally (OECD/FAO, 2021). Due to global population growth (Taagepera and Nemčok 2024), the demand for pork is expected to rise, necessitating technological innovations that enhance its sustainable production, with intensive farming being the most efficient approach. Intensive pork production, however, is challenged by issues related to animal welfare and various stressors that pigs encounter that are part of routine husbandry practices, including noninfectious stressors, such as weaning, transportation, agonistic social interactions, human handling, heat or cold, etc., as well as by infectious stressors that can result in disease. Both infectious and noninfectious stress have been shown to compromise productivity, inhibit growth, and stimulate inflammatory response pathways (Dobson and Smith 2000; Prunier *et al.* 2005; Gimsa *et al.* 2018; Rohleder 2019), resulting in complex and multifaceted changes in the animals.

Under conditions perceived to be stressful, an individual acutely responds via activation of the sympathetic nervous system (Joëls and Baram 2009), which is rapidly followed by activation of the hypothalamic–pituitary–adrenal (HPA) axis, leading to a series of physiological and behavioral adaptations that are aimed at achieving homeostasis and increasing the chances of survival. This involves the release of corticotropic releasing hormone from the hypothalamus, followed by production of the adrenocorticotropic hormone (ACTH) from the anterior pituitary, and finally the production of glucocorticoids such as cortisol from the zona fasciculata layer of the adrenal cortex into the blood circulation above levels that are produced during normal circadian rhythms (Nicolaides *et al.* 2014; Angelousi *et al.* 2020; Kazakou *et al.* 2023). Glucocorticoids are thus the final hormonal effectors of the HPA axis (Tsigos *et al*. 2020) and are essential for homeostasis and for many vital physiological functions such as growth, immune response, reproduction, and cell proliferation and metabolism (Charmandari *et al.* 2005). Most cortisol circulates in an inactive form, bound to the corticosteroid-binding globulin or albumin (Lewis *et al.* 2005; Hammond 2016), and is activated by isoform 1 of the 11-beta-hydroxysteroid dehydrogenase enzyme (11-β-HSD1). Cortisol can also be converted to cortisone, by isoform 2 of the 11-beta-hydroxysteroid dehydrogenase enzyme (11-β-HSD2), which has marginally reduced glucocorticoid activity (Schiffer *et al.* 2019; Thau *et al.* 2023).

During physiological stress response, dehydroepiandrosterone (DHEA) and its sulfate form (DHEA-S) are also produced. DHEA and DHEA-S are anabolic steroids that are produced by the zona reticularis layer of the adrenal cortex, but also in the brain, testes, and ovaries, but will be referred to as stress hormones within the context of this study because of their regenerative roles to counteract the physiological effects of glucocorticoids by having immune-enhancing properties (Hechter *et al.* 1997; Maninger *et al.* 2009; McNelis *et al.* 2013; Whitham *et al.* 2020). DHEA is enzymatically produced from cholesterol and can then be sulfated to a more stable form (DHEA-S) through a process catalyzed by hydroxysteroid sulfotranferase (HST, SULT2A1). DHEA-S can be desulfated to DHEA in almost all parts of the body, a process catalyzed by the steroid sulfatase (STS) enzyme. About 98% of this hormone in circulation exists as DHEA-S (Kamin and Kertes 2017).

DHEA and DHEA-S are often used interchangeably in literature and will be referred to as DHEA(S) in this article, although there is some evidence to show that they are different. For example, the blood levels of DHEA exhibit episodic diurnal fluctuations, while those of DHEA-S fluctuate significantly less throughout the day (Rosenfeld *et al.* 1975). DHEA-S binds more strongly to albumin, its protein carrier, than DHEA, which contributes to its slower metabolic clearance. Furthermore, in events where the 2 hormones control a similar process such as increasing dopamine levels under stress, the mechanisms with which they achieve this are different (Tomas-Camardiel *et al.* 2002; Charalampopoulos *et al.* 2005; Maninger *et al.* 2009; Gowrishankar *et al.* 2014; Pérez-Neri *et al.* 2020).

Stress hormones are incorporated into hair as it grows through various hypothesized processes (Henderson 1993; Russell *et al.* 2012; Stalder and Kirschbaum 2012). Their concentration in hair, therefore, reflects an integrated index of HPA axis activity, offering a retrospective assessment of stress response over weeks or months (Russell *et al.* 2012; Burnard *et al.* 2016). In addition, ratios of stress hormones that are assumed to coregulate each other represent complex interactions that may represent the physiological environment better than levels of the individual hormones (Sollberger and Ehlert 2016).

Against this background, we hypothesized that the levels of stress hormones and their ratios in hair of young and clinically healthy pigs are genetically determined and can thus be modified by genetic selection, which can make them vital tools for selecting pigs that respond differently to noninfectious stressors that are part of routine management in pork production. We also hypothesized that the levels of these stress hormones in hair of young and clinically healthy pigs are genetically related to the coping response style of pigs to noninfectious stressors.

The first objective of this study was to investigate the genetic basis of levels of stress hormones and their ratios in hair of young and clinically healthy pigs. The second objective was to assess their potential as genetic biomarkers of the pigs’ innate coping response style to noninfectious stressors by estimating their genetic correlations with responses to the backtest as a validated method of assessing the innate coping response style of pigs (Hessing *et al.* 1993; Bunter and Lansdowne 2004; Melotti *et al.* 2011). We then performed genome-wide association studies (GWAS) to identify quantitative trait loci (QTL) that drive the genetic variance in the levels of stress hormones in hair and in backtest responses, followed by gene ontology (GO) enrichment analyses for genomic regions that did not reach significance in the GWAS, as suggested by Holden *et al.* (2008), in order to uncover biological processes that are associated with the genetic control of stress hormone levels and backtest responses. Results from this study can facilitate the selection of pigs in high-health nucleus breeding programs that cope better with noninfectious stressors in commercial settings, using a relatively noninvasive approach. Subsequent studies will evaluate the potential of these measures of stress response in young and clinically healthy pigs as genetic biomarkers of response to infectious stressors and, therefore, disease resilience.

## Methods

### Animals used

This study was part of the Natural Disease Challenge Model (NDCM) described by Putz *et al.* (2019) and Cheng *et al*. (2020). The stress response data analyzed were from 15 batches of 60 or 75 Yorkshire × Landrace F1 castrated males (barrows) from 7 breeding companies, members of PigGen Canada (Alliance Genetics, AlphaGene, DNA Genetics, AcuFast, Genesus, Hypor, and Topigs-Norsvin). These batches were entered into the NDCM subsequent to the batches that were analyzed by Putz *et al.* (2019) and Cheng *et al.* (2020). Each batch consisted of pigs from 1 breeding company and originated from 1 healthy multiplier farm. A new batch was entered into the quarantine nursery of the NDCM every 3 weeks, with pigs randomly allocated to 1 of 4 or 5 pens. All data used for this study were collected in the quarantine nursery. Piglets in each batch were progeny from at least 9 boars, with overlap in sires between batches from the same company. The number of barrows submitted per litter was 2, 3, or 4, providing an adequate structure to estimate genetic parameters and litter effects, while representing the genetic diversity within the maternal nucleus breeding populations of each company. Because of limited budget and labor availability, the total number of animals evaluated (889) in this study was at the lower end for some of the genetic analyses conducted. However, because of the novelty of the traits evaluated, results will be presented and discussed with somewhat more lenient significance thresholds.

### Genotypes

While pigs from the original 50 batches of the NDCM described by Cheng *et al.* (2020) were genotyped using a 650 K single nucleotide polymorphism (SNP) panel, with quality control as described by Putz *et al.* (2019), animals from the additional 15 batches evaluated here were genotyped using a custom 50 K Affymetrix SNP panel by Eurofins Scientific. Using the 650 K genotypes of the earlier batches, which consisted of animals from the same companies and lines, these 50 K genotypes were imputed up to 650 K using FImpute (Sargolzaei *et al.* 2014), separately for each company. The final genotype file included genotypes for 451,343 markers that mapped to 19 chromosomes of the *Sus scrofa* (SSC) genome. Genomic regions that were found to be strongly associated with traits of interest based on GWAS were imputed to whole-genome sequence (WGS) using the SWine IMputation (SWIM) 1.0 public web server (https://quantgenet.msu.edu/swim/), which uses a reference haplotype panel that was developed based on WGS data on 2,259 animals, representing 44 pig breeds, as described by Ding *et al.* (2023).

### Phenotypes

#### Stress hormone levels in hair

Hair was shaved from the hip area of all 863 piglets from the 15 batches at the end of the quarantine nursery period, at ∼40 days of age. The aim here was to capture hair grown from birth to ∼3 weeks postweaning and assess the corresponding stress hormone levels, in response to stressors that the pigs were exposed to during that time. Watson and Moore (1990) showed that the first hair and growth phase is complete at birth in the pig.

Extraction and analysis of hair hormones was conducted at the University of Saskatchewan, following the standardized and validated protocols described in Creutzinger *et al.* (2017) and Casal *et al.* (2017). On average, 123 mg of hair from each pig was washed in 0.04 mL of solvent (methanol) per mg of hair for 3 min on a slow rotator and patted dry on a paper towel. This procedure was repeated 2 more times. Washed hair was then dried for 24 h at room temperature. Samples were ground using a Retsch MM 301 Mixer Mill at ∼0.03 min/mg of hair at 30 Hz to achieve a fine powder. Ground samples were stored in microcentrifuge tubes, boxed to protect from light for a varying number of days before extracting the hormones (Supplementary Fig. 1). The effect of storage length was fitted as a fixed covariate in statistical models for all hormone traits evaluated.

For each hormone measured, 25 mg of powdered hair was incubated in 0.5 mL of methanol for 24 h on a slow rotator and centrifuged for 15 min (2,150*×g* at 20°C). The supernatants were evaporated at 38°C under nitrogen gas for 45 min. The samples were rinsed with 1.5 mL of methanol, vortexed, and centrifuged twice. Tubes were rinsed with decreasing volumes of the solvent (0.8, 0.6, and 0.4 mL), evaporated under nitrogen gas, and reconstituted with 0.4 mL of assay diluent buffer (12 h at 4°C) provided with the ELISA kits and thereafter frozen at −80°C until analysis. The reconstituted samples in assay buffer were analyzed via 2 ELISA kits in accordance with the manufacturers’ guidelines: the Arbour Assays (Ann Arbour, MI, USA) for cortisone, and commercial ELISA kits (Salimetrics, LLC, Carlsbad, CA, USA) for the other 3 hormones. The commercial ELISA kits were validated for cortisol and DHEA but not for DHEA-S but appeared to perform well for the latter also. Based on the assay results, the concentration of a hormone (pg/mg) in hair was computed as $\;\frac{S\times R_{v}}{E_{m}\;}d_{f}$, where S is the mean assay result in pg/ml for the duplicate assays for a given pig, $R_{v}$ is the reconstitution volume in ml, $E_{m}$ is the extraction mass in mg, and $d_{f}$ is the dilution factor. Prior tests on a set of pooled samples were used to determine the dilution factor for each hormone. As a result, 197 DHEA samples were diluted with $d_{f}=8$, and 674 samples with $d_{f}=16$. For DHEA-S, $d_{f}=2,\;3,\;4,\;8,\;\mathrm{and}\;16$ were used for 6, 3, 123, 690, and 1 sample, respectively. The $d_{f}$ values used for cortisone samples fell on a continuous scale ranging from 2 to 4, while cortisol samples were not diluted.

Cortisol, DHEA, and DHEA-S were analyzed in duplicate and, hence, the concentration for a sample was obtained as the average of the duplicates. The cortisone assay, however, persistently produced high within-sample coefficients of variation (CV) and was, therefore, analyzed in triplicate and the assay result was initially obtained as the average of the 2 replicates that produced the lowest within-sample CV. Retrospective analyses found no substantial difference in estimates of heritability for cortisone when based on the 2-replicate average vs on the average of triplicates. In addition, estimates of phenotypic and genetic correlations for cortisone based on these 2 sets of averages were very high (0.89 ± 0.01 and 0.92 ± 0.36, respectively). Thus, analyses were not repeated for the 3-replicate average and results based on the 2-replicate averages will be presented for cortisone.

Nine samples for cortisone and 30 samples for DHEA-S had CV outside their respective acceptable ranges. The assay could, however, only be repeated for 2 of the 9 samples for cortisone due to insufficient sample remaining but these still resulted in high within-sample CV. For DHEA-S, the assay was repeated for 19 of the 30 samples due to limited sample remaining, but these repetitions also still had a high CV. Including or eliminating the samples with high CV from the data did, however, not significantly affect estimates of genetic variance for the respective traits and, hence, all these samples were included in the final dataset for analysis. Additional data quality checks involved identifying and eliminating outliers using a modified interquartile range (IQR) method (Vinutha *et al*. 2018). An outlier was defined as any observation above the upper boundary (Q3 + *k* * IQR), where Q3 is the third quartile, IQR is the interquartile range, and *k* is a constant. The latter is typically set to *k* = 1.5 (Vinutha *et al*. 2018) but 4.5 was used here such that the method would be consistently applied to all hormones while retaining most of the data points. Three samples with cortisol > 54.9 pg/mg, 5 samples with cortisone > 96.4 pg/mg, and 1 sample with DHEA > 42.5 pg/mg were eliminated from the analyses, while there were no outliers for DHEA-S concentration based on this criterion.

In addition to the level of each individual hormone, we also evaluated the sum of the levels of glucocorticoids (SOG = cortisol + cortisone) and the sum of DHEA and DHEA-S (SOD). Because of the novelty of the data collected, ratios of the different hormones were also evaluated, including SOG/SOD. Ratios were computed using the sample-average concentrations of the respective hormones, after removing outliers.

#### Backtest responses

The backtest was performed on all pigs at ∼27 days of age, 5 days after their arrival in the quarantine nursery to allow for acclimation to the new environment and pen mates. Following protocols described by Hessing *et al.* (1993) and Melotti *et al.* (2011), each pig was placed in a supine position in a V-shaped restrainer that was placed on a panel on the top of the adjacent pen, with the tail toward the pen and the head toward the wall and lightly restrained with 1 hand over the thorax and abdomen. Recording of responses to this stressor began as soon as the pig was placed on its back, including the intensity and number of vocalizations (VI and VN) and of struggles (SI and SN) and continued for 30 s. Although many studies perform this test for 1 min (Spake *et al.* 2012; Zebunke *et al.* 2015; Iversen *et al.* 2017), 30 s was used here to reduce labor requirements and because pigs were somewhat larger in size, which made it hard to handle the pigs once they started to struggle vigorously.

Vocalizations were counts of continuous noise made by the pig and could involve screams or grunts that ranged in pitch and volume. An individual vocalization was counted when there was a break in the noise between emitting 1 vocalization and then starting another. A struggle was classed as 1 bout of continuous movement (thrashing of the limbs back and forth, sometimes accompanied by twisting of the thorax and abdomen). A pause in movement for more than 2 s, followed by resumed movement was then considered a new struggle bout. The intensities of struggling and vocalizations were subjectively scored on a scale from low to high, as described in Table 1. Backtest responses were simultaneously evaluated by 2 experienced technicians, with the technician holding the piglet registering SN and SI and the other registering VN and VI. Across the 15 batches, 5 and 6 technicians were used record vocalizations and struggles, respectively. The same technician recorded all pigs in a given batch for a given trait. The effect of the technician was, therefore, confounded with the effect of batch.

**Table 1.** Description of scores assigned to the intensity of vocalization and struggles during the 30 s backtest.

Score	Struggles intensity	Vocalization intensity
1	Without struggles (very quiet, no movement)	No noise
2	Weak attempt to escape (slow movement of the limb)	Quiet noise (grunting requiring attentive listening to notice)
3	Medium strength to escape (rapid movement of the limb)	Noisy (clearly audible by the observer)
4	Strong attempt to escape (participation of the head and column vertebra)	Loud (audibly uncomfortable scream)
5	Very strong attempt to escape (difficult to keep pig in supine position during the test)	

### Statistical analyses

#### Variance component estimation

**Fig. 1.**
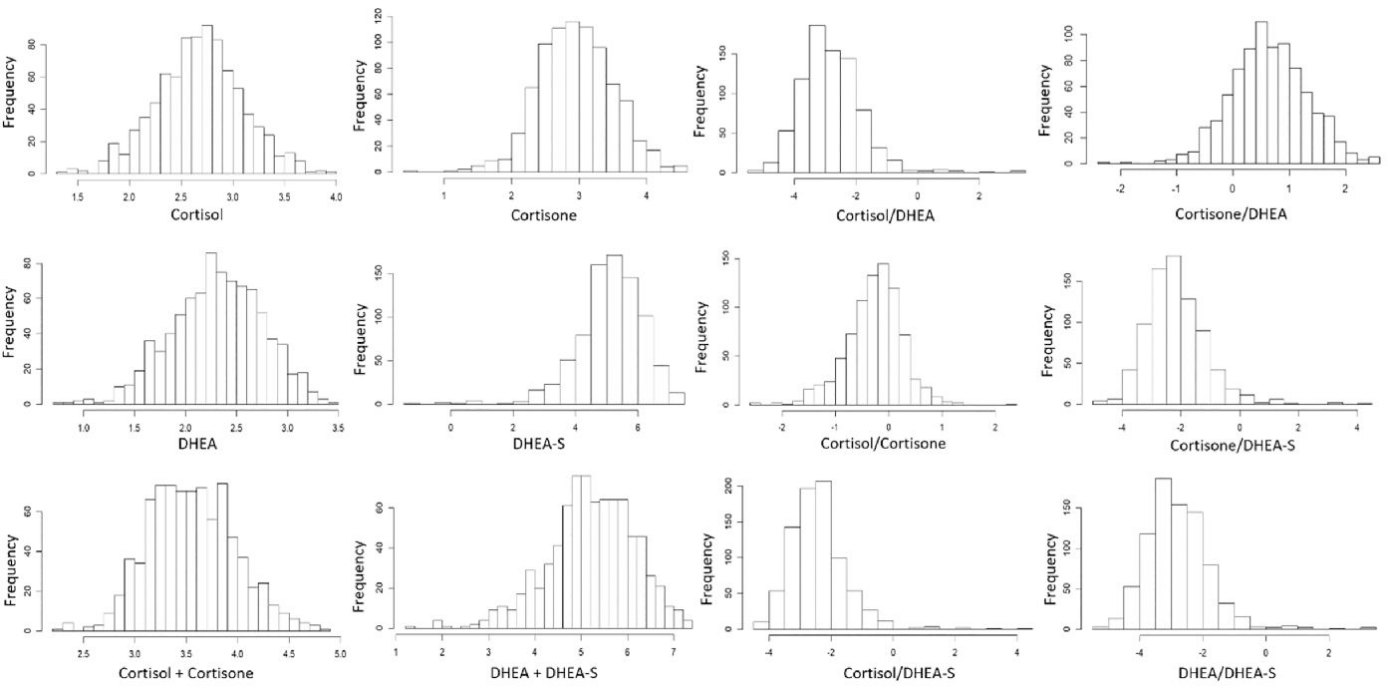
Distributions of natural log-transformed levels of stress hormones and of their ratios in hair of 863 young and clinically healthy pigs.

Kolmogorov–Smirnov tests indicated that the concentrations of the stress hormones and their ratios did not follow a Gaussian distribution and hence the levels of all hormones (including SOG and SOD) and their ratios were transformed using the natural log for subsequent statistical analyses (Fig. 1). Variance components and genetic correlations were estimated by generalized linear mixed models with a genomic relationship, using ASReml 4.2 (Gilmour *et al.* 2021). Estimates of heritability were obtained using linear mixed models for all stress hormones. Lagrange multiplier tests using functions in the R package *AER* v.1.2-14 (Kleiber and Zeileis 2008) showed that VN and SN were significantly over-dispersed (*P* < 0.001), with dispersion values of 6.3 and 2.5, respectively. Thus, a negative binomial model was used to estimate their variance components, while cumulative multinomial models were used to analyze VI and SI. Due to computational limitations, all bivariate analyses were conducted using linear mixed models. For the number of vocalizations and struggles, log(1 + count) was used in their bivariate analyses. The general structure of the model fitted was:


(1) $$y_{ijkl\;}=\mathrm{batc}h_{i\;}+\mathrm{ag}e_{ijkl\;}+\mathrm{pe}n_{\;j\;}+\mathrm{litte}r_{ijk}+\;u_{ijkl\;}+e_{ijkl\;}$$

where $y_{ijkl\;}$ is the trait phenotype. Fixed effects included $\mathrm{batc}h_{i}$ (for i=1,2,..15) and the covariates of $\mathrm{ag}e_{ijkl\;}$ at entry into the quarantine nursery. For stress hormone levels, length of $\mathrm{storag}e_{ijkl\;}$ of the ground hair samples before hormone extraction was fitted as another covariate. Random effects included animal genetics ($u_{ijkl\;})$), residual effects ($e_{ijkl\;})$), as well as $\mathrm{pe}n_{\;j\;}$ and $\mathrm{litte}r_{ijk}$ effects if they explained a substantial amount of variation for the trait analyzed. As a result, the litter effect was eliminated from the models for SN and VN, while the pen effect was eliminated from the models for cortisol and VI. Vectors of pen and litter effects were assumed distributed $pen\;∼\;N(0,\;I\sigma_{p}^{2}),\;$ where I is an identity matrix and $\sigma_{p}^{2}$ is the pen variance, and $litter\;∼\;N(0,I\sigma_{l}^{2}),$ where $\sigma_{l}^{2}$ is the litter effects variance. The vector of animal genetic effects was assumed distributed $u\;∼\;N(0,G\sigma_{A}^{2}),$ where G is the genomic relationship matrix among animals and $\sigma_{A}^{2}$ is the additive genetic variance. Using the imputed 650 K genotypes, separate genomic relationship matrices were constructed for each company using preGSf90 (Misztal *et al.* 2002) based on method 1 of VanRaden (2008) and then combined into an overall G matrix with relationships between companies set to zero to obtain pooled estimates of within company genetic variances, as described by Cheng *et al.* (2020). The vector of residual effects was assumed distributed $e\;∼\;N(0,\;I\sigma_{e}^{2}),$ where $\sigma_{e}^{2}$ is the residual variance. For stress hormones, additional independently and identically distributed random effects fitted included hair grinding date, with 55 levels, and extraction date, with 19 levels for DHEA-S and 22 levels for cortisol, cortisone, and DHEA.

Estimates of heritability and of the proportion of variance due to litter were calculated as ratios of the estimates of variance due to additive genetic and litter effects, respectively, to the total phenotypic variance, which was the sum of the estimates of additive genetic variance, litter variance, and residual variance. For bivariate analyses, estimates of genetic correlations were computed as the ratio of estimates of genetic covariance to the product of estimates of the genetic standard deviation for the 2 traits. Significance of deviations of genetic correlation estimates from 0 or 1 were assessed using a likelihood ratio test for models with and without the genetic correlation set to 0 or 1, respectively. The *P*-values were then obtained by comparing the test statistic to a χ^2^ distribution with 1° of freedom.

### Genome-wide association study

The univariate form of the marker-based BayesB model (2) described below (Habier *et al.* 2011) was used to detect QTL for each trait, while the bivariate form of the model was used to detect pleiotropic QTL for pairs of traits. Marker effects were estimated by fitting the genotypes of all SNPs from the imputed 650 K panel simultaneously as random effects using Bayesian variable selection methods (Cheng *et al.* 2018). The general structure of the model used was:


(2) $$y_{ijkl\;}=\mathrm{batc}h_{i\;}+\mathrm{ag}e_{ijkl\;}+\mathrm{pe}n_{\;j\;}+\mathrm{litte}r_{ijk}+\;\sum_{n=1}^{p}\;m_{ijkln}\beta_{n}\delta_{n}+e_{ijkl\;}$$

with fixed and random effects defined as for model (1), except with animal additive genetic effect fitted as the sum of SNP allele substitution effects instead of as animal breeding values. For model (2), $m_{ijkln}\;$ is the genotype for SNP *n* (coded as 0, 1, and 2) for a total of *p* SNPs (p=451343)), with allele substitution effect $\beta_{n}\delta_{n}$, where $\delta_{n}$ is an indicator whether SNP *n* was included ($\delta_{n}=1$) in the model or not ($\delta_{n}=0$) for a specific iteration of the Monte Carlo Markov Chain that was fitted; effects $\beta_{n}$ were assumed to be independently distributed as $\beta_{n}\;∼\;N(0,\sigma_{n}^{2})$, where $\sigma_{n}^{2}$ is the locus-specific marker effect variance, which was assumed to follow a scaled inverted χ^2^ distribution with scale parameter $S_{\beta }$ and degrees of freedom $v_{\beta }$. The prior probability that a SNP has zero effect (*π*) was estimated using BayesC*π* (Habier *et al.* 2011) and ranged from 0.997 to 0.999 but for consistency, *π* = 0.999 was used for all univariate GWAS using the BayesB method.

Bivariate analyses for pairs of traits were also performed using model (2), with the same fixed effects as in the respective univariate analyses. In the bivariate models, the vector of random effect, *i*, $R_{i}$ (size 2) of pen, litter, and residual for the pair of traits were assumed to be distributed bivariate normal, $R_{i}∼BVN(0,U_{i})$, for $U_{i}=\left[\begin{array}{cc} \sigma_{{⋃}_{i}1}^{2} & \sigma_{{⋃}_{i}1,2}\\ \sigma_{{⋃}_{i}2,1} & \sigma_{{⋃}_{i}2}^{2} \end{array}\right]$, where $U_{i}$ is the variance-covariance matrix of random effect *i*, assumed to have an inverse Wishart prior distribution, with scale parameters $S_{i}$ and degrees of freedom $v_{i}$. For a given SNP *n*, the effects on the 2 traits were modeled as $D_{n}\beta_{n}$, where $\beta_{n}$ is the vector with effects on the 2 traits and $D_{n}=\left[\begin{array}{cc} \delta_{n1} & 0\\ 0 & \delta_{n_{2}} \end{array}\right]$, where $\delta_{n1},\;\delta_{n2}$ indicate whether SNP *n* was included in the model with nonzero effects for trait 1 and/or trait 2 in a given iteration of the chain. Thus, $D_{n}$ had 4 possible combinations for the diagonal elements, that is $\left[\delta_{n1},\;\delta_{n2}\right]$: [0, 0], [0, 1], [1, 0], and [1, 1], with assumed prior probabilities equal to 0.999^2^, 0.999(1–0.999), (1–0.999)0.999, and (1–0.999)^2^, respectively. Vector $\beta_{n}$ was assumed to be distributed bivariate normal, $\beta_{n}∼BVN(0,G_{n})$ for each SNP, with independence between SNPs, where $G_{n}$ is a locus-specific variance-covariance matrix, $G_{n}=\left[\begin{array}{cc} \sigma_{\beta_{n1}}^{2} & \sigma_{\beta_{n1,2}}\\ \sigma_{\beta_{n2,1}} & \sigma_{\beta n2}^{2} \end{array}\right]$, which was assumed to have an inverse Wishart prior distribution with degrees of freedom $v_{\beta }+1$ and mean $S_{\beta }$ + $\beta_{n}\beta_{n}^{\prime }$, where $S_{\beta }$ is a scale parameter. The univariate and bivariate GWAS were performed using the BayesB method implemented in the JWAS package (Cheng *et al.* 2018), with a chain length of 80,000, discarding the first 5,000 iterations as burn-in. Convergence was evaluated by visual inspection of trace plots of the sampled parameters.

A QTL identified by the univariate GWAS was defined as a nonoverlapping 1-Mb window that explained more than 1% of the estimated genetic variance (EGV) for the trait, computed as described by Wolc *et al.* (2012). Pleiotropic QTL were detected using the posterior probability of a genomic window having a nonzero covariance, as obtained from the bivariate GWAS. For this, considering windows as large as 1 Mb increases the probability that, for a given window, a given iteration of the chain includes both SNPs for which the sampled allele substitution effects for the 2 traits have the same and opposite signs, resulting in positive and negative covariances cancelling each other out in the window, which reduces the overall window covariance, increases false negatives, and reduces power to identify pleiotropic regions. To reduce this effect, 0.25 Mb nonoverlapping windows across the genome were used instead. At first, the posterior probability that a window had a nonzero covariance (PP_cov) for the pair of traits was proposed as a measure of pleiotropy. However, this tended to be affected by the number of SNPs in the window, as windows with more SNPs tended to have a higher PP_cov than windows with fewer SNPs, regardless of the magnitude of the covariance explained by the SNPs. Instead, for each 0.25 Mb window, the absolute difference (Ad) in the posterior probability that a window had a positive (PP_pos) vs a negative covariance (PP_neg) was computed and used to identify pleiotropic QTL if Ad was greater than an arbitrary threshold of 0.02.

### Fine mapping of QTL

Within an identified QTL window, the imputed 650 K SNPs were used to impute the region to WGS using the public SWIM server (Ding *et al*. 2023) and GWAS was then repeated using the imputed genotypes. Genotypes of the most significant SNP in a QTL window (lead SNP) were fitted as a fixed covariate in the univariate form of model (1) to determine the significance of its association with a trait and to estimate its allele substitution effect for the trait. The lead SNPs were also fitted as a fixed covariates in the univariate form of GWAS model (2) to determine the amount of genetic variance at the QTL they removed.

To determine the breed origin of the QTL, the 1 Mb region around the imputed lead SNP was phased using SWIM server (Ding *et al.* 2023) and haplotypes were compared to those identified in animals from the Landrace and Yorkshire breeds that are part of the reference population used by Ding *et al.* (2023). Then, genotype at the lead SNP was fitted using 2 fixed covariates, 1 for each breed origin to estimate their effects, depending on breed origin.

Linkage disequilibrium (LD, based on *r*^2^) between markers was estimated using the Haploview 4.2 software (Barrett *et al.* 2005). Due to the high LD between SNPs, a single lead SNP could not be identified as a causal variant with enough statistical confidence. Hence, if a SNP was found to explain a considerable amount of the QTL effect, other SNPs that had LD ≥ 0.4 with it were also considered potential causal variants, and their predicted variant effects were obtained using the Ensembl Variant Effect Prediction (VEP) tool (McLaren *et al.* 2016).

### Candidate genes in QTL

Pocrnic *et al.* (2024) showed that, in typical pig breeding populations, because of the extent of LD, 80% of the genetic variance that is captured by an identified QTL lies within a 5 Mb window around the QTL. Thus, to identify positional candidate genes for the identified QTL regions, genes within a region of 5 Mb centered around an identified 1 Mb QTL window were identified with BioMart (Smedley *et al.* 2009), using the Ensembl pig gene database (*Sscrofa*11.1). A gene was declared a candidate based on its annotated functional relationship with the trait of interest, as supported by existing literature.

To investigate whether the identified SNPs affected the expression of candidate genes within QTL regions, population-level gene transcript abundance data in blood of 861 of these pigs were used. The expression data were obtained as described by Lim *et al.* (2023) from blood samples collected at ∼27 days of age in the quarantine nursery of the NDCM by 3′mRNA sequencing with a globin block. After sequence alignment, reads were normalized across all samples by trimmed M values, while excluding those that mapped to the globin genes *HBA* and *HBB* and that had zero counts in more than 80% of samples. Scaled gene expression values were obtained by a log2 transformation of the normalized counts plus 1. Using the resulting expression values of a candidate gene as the response variable, genotypes of the lead SNPs in the QTL were fitted as a fixed covariate in the following univariate linear mixed model:


(3) $$\begin{array}{rl} y_{ijklm\;}= & \;{\mathrm{batch}}_{i}\;+{\mathrm{age}}_{ijklm}\;+\underset{\begin{array}{c} cell\\ type\;c \end{array}}{\sum }\;{\mathrm{COMP}}_{cijklm}+{\mathrm{pen}}_{\;j}\;+{\mathrm{litter}}_{ijkl}\\ & +\;u_{ijklm}\;+e_{ijklm} \end{array}\;$$

where COMP*_cijklm_* represents the covariate of the log2 of the proportion of white blood cell type *c* (*c* = lymphocytes, neutrophils, monocytes, basophils, eosinophils, and large unstained cells) based on complete blood counts, as described by Lim *et al.* (2023) and other effects were as described for model (1).

### Gene set enrichment analyses

Although the GWAS identified some QTL, other regions that explain smaller proportions of the genetic (co)variance may have registered as false negatives because of the limited statistical power of GWAS. To understand the biological relevance of these nonsignificant regions of the genome, a ranked gene set enrichment analysis (GSEA) was performed using the GSEA software (Subramanian *et al.* 2005). The GO library used was developed as described by Cheng *et al.* (2022), by assigning GO terms to each 0.25 Mb window based on the genes that are present in that window and their annotation in the Molecular Signatures Database (MSigDB, c5.all.v7.0.-symbols.gmt; Subramanian *et al.* 2005). Terms that were assigned to fewer than 11 or to more than 8,999 windows were not included in the library. The 0.25 Mb windows were ranked by the %EGV they were estimated to explain for a given trait, after excluding windows that were significant based on the GWAS (with >1% EGV), as the aim was to identify biological processes that are enriched among genomic regions that were estimated to explain genetic variance for the trait but not at a level to declare significance. For enrichment analyses of pleiotropic regions, 0.25 Mb windows were ranked based on their Ad values for the pair of traits of interest.

To increase power and allow better interpretation of the results of the enrichment analyses, traits were grouped for joint GSEA of glucocorticoids (cortisol and cortisone), all heritable stress hormones (cortisol, cortisone, and DHEA-S), both struggle traits (SI and SN), both vocalization traits (VI and VN), and all backtest response traits (SI, SN, VI, and VN). For enrichment analysis of a group, the ranked 0.25 Mb windows for each trait (based on the %EGV of each window for a given trait) were combined and reranked, as described by Bhatia *et al.* (2023) . A similar approach was used for joint GSEA of windows based on pleotropy, ranked by Ad values for each pair of traits.

## Results and discussion

We present the first study on the genetics of levels of stress hormones and their ratios in hair of pigs and assess the potential of hair collected on young and clinically healthy pigs to inform on the response style of pigs to noninfectious stressors by estimating their genetic and phenotypic relationships with responses to the standard backtest (Bunter and Lansdowne 2004). The use of stress hormones in hair as a noninvasive retrospective assessment of stress response in clinically healthy young animals would be of high value in swine breeding programs, especially because the breeding pyramid requires selection candidates to be raised in disease-free environments and selection is preferably performed when animals are still young (Knap 2005; Knap and Doeschl-Wilson 2020). In this study, hair was collected from pigs at ∼40 days of age to capture stress hormones that had accumulated during response to any noninfectious stressors that they encountered up to that point of their lives. Although stressors that the pigs were exposed to may have differed between batches, pigs within a batch were born within a couple of days of each other, in the same multiplier farm, transported together, and housed together in the quarantine nursery. As a result, pigs within a batch were exposed to very similar stressors, allowing genetic differences in response to these stressors, as reflected in levels of hair hormones in hair, to be ascertained. The multiplier farms that the pigs originated from had high levels of biosecurity and tested negative for major pathogens. In addition, all piglets were evaluated to be clinically healthy upon entry into the quarantine nursery, which was also maintained free of major pathogens. Thus, up to the point when the hair was collected, piglets were not exposed to major diseases and, thus, the effects of stressors on the levels of stress hormone evaluated were primarily the result of responses to noninfectious rather than infectious stressors. Subsequent study will evaluate responses to infectious stressors of these same pigs, as part of the NDCM, including stress hormone levels in hair grown during exposure to infectious stressors, as well as associated disease resilience traits in terms of performance and clinical disease traits. Although selection programs in pigs operate on purebred populations, the pigs evaluated in this study were crossbreds of 2 of such purebred populations because pigs that are raised for commercial pork production are predominantly crossbreds.

### Biological description of the data

Figure 1 shows the distributions of natural log-transformed hormone traits in hair of the evaluated pigs, while Table 2 shows their summary statistics. The average level of cortisone (the inert glucocorticoid) was higher than that of cortisol (the active glucocorticoid), possibly because cortisone is less polar than cortisol and hence may have better incorporation into hair from the bloodstream (Raul et al. 2004), but we could not verify this. It could also indicate higher activity of type 2 than type 1 of the 11-β-HSD enzyme during this phase of hair growth. In humans under the age of 16, the type 2 isozyme of 11-β-HSD is more active than type 1 in the brain, skin, gastrointestinal tract, and gonads, mainly to prevent the negative effects of cortisol on growth by converting it to cortisone (Raul *et al.* 2004). The same could be the case for young pigs, given that pigs and humans have very similar stress response systems (Gimsa *et al.* 2018), but this could also not be validated since it requires comparison with older pigs under similar circumstances. On the other hand, the average level of DHEA-S in hair was higher than that of DHEA. DHEA-S is generally more stable than DHEA, as sulfation makes it less prone to rapid metabolism or degradation. It was the most abundant hormone of those evaluated and could indicate that hydroxysteroid sulfotransferase (HST or SULT2A1) was more active than STS during this phase of a piglet's life.

**Table 2.** Summary statistics and the effects of storage time (days) on the concentrations of stress hormones and their ratios in the hair of young healthy pigs, and backtest responses.

								Storage effect	
Traits evaluated	No. of pigs	Mean	SD	Median	Min	Max	CV (%) Inter/Intra	Estimate (SE)	*P*-value
Hormones (pg/mg)									
Cortisol	860	15.8	6.8	14.5	3.4	49.4	14.5/5.8	−0.002 (0.002)	0.18
Cortisone	830	22.4	13.1	18.7	3.1	91.9	15.5/13.5	0.003 (0.006)	0.58
DHEA	862	11.1	4.9	10.2	2.1	31.8	15.7/10.4	0.001 (0.002)	0.58
DHEA-S	815	257.6	240.2	178.3	0.2	1428.5	12.87/13.2	−0.003 (0.002)	0.24
Cortisol + Cortisone (SOG)	827	38.2	17.3	34.0	9.9	123.0		−0.009 (0.004)	0.71
DHEA + DHEA-S (SOD)	812	268.8	242.0	189.1	3.7	1437.0		−0.003 (0.004)	0.07
Hormone ratios									
Cortisol/DHEA	858	1.6	0.8	1.4	0.2	8.2		−0.003 (0.002)	0.21
Cortisol/Cortisone	827	0.6	0.4	0.8	0.1	10.7		−0.004 (0.003)	0.13
Cortisone/DHEA	830	2.4	1.8	1.8	0.1	13.3		−0.001 (0.003)	0.68
Cortisol/DHEA-S	811	0.3	2.4	0.1	0.0	61.9		−0.009 (0.004)	0.03
Cortisone/DHEA-S	798	0.4	3.0	0.1	0.0	76.7		0.010 (0.005)	0.05
DHEA/DHEA-S	812	0.2	1.3	0.1	0.0	28.4		−0.005 (0.004)	0.50
SOG/SOD	795	0.3	0.4	0.2	0.0	6.8		−0.006 (0.004)	0.50
SOG/DHEA-S	796	0.6	5.4	0.6	0.0	138.6		−0.030 (0.060)	0.61
Backtest									
Vocalization number	889	5	6	3	0	28			
Vocalization intensity	889	2	1	2	1	4			
Struggle intensity	889	3	1	3	1	5			
Struggles number	889	3	3	2	0	16			

Abbreviations: CV, coefficient of variation; SD, standard deviation; SE, standard error.

**Fig. 2.**
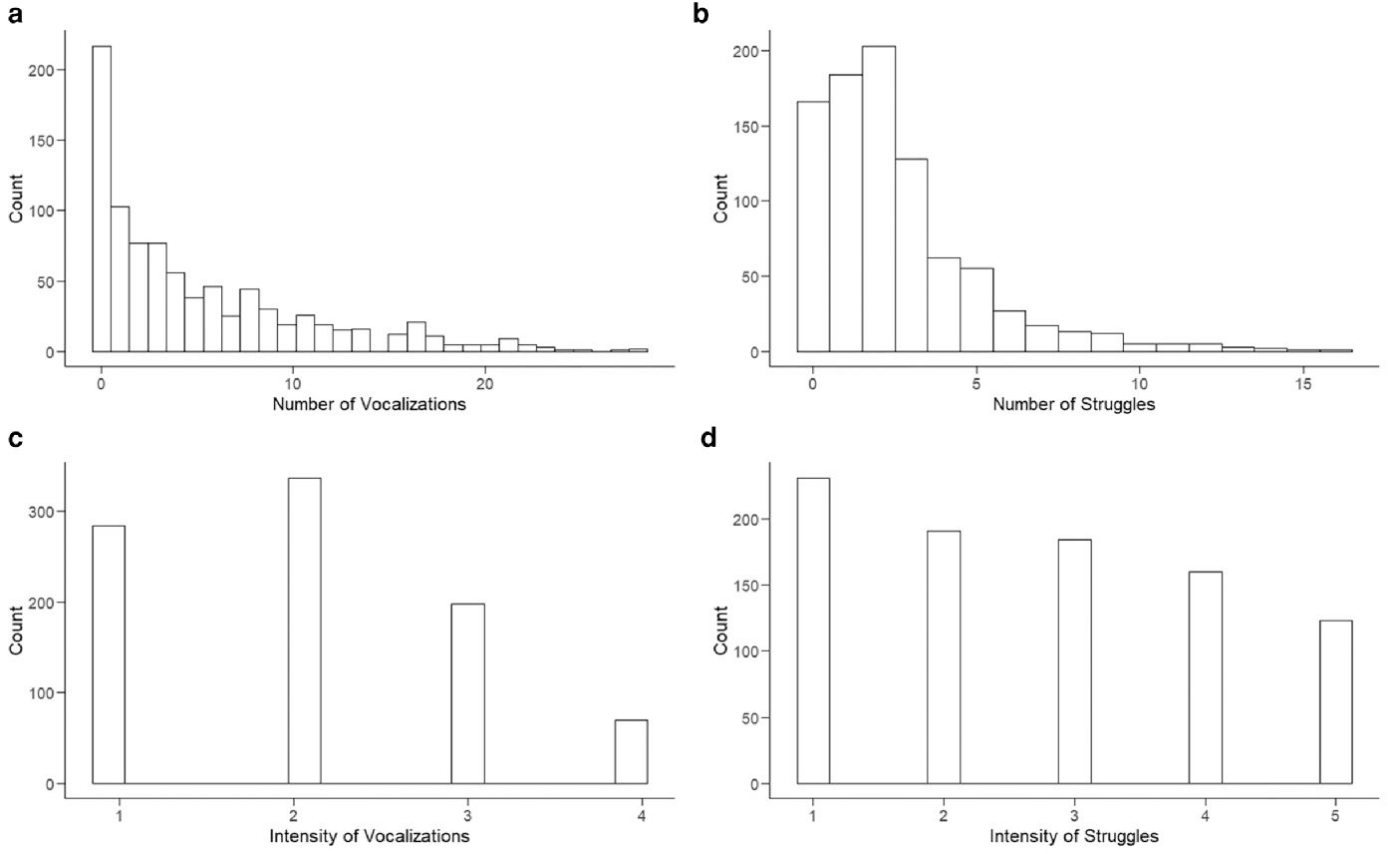
Distribution of the number of a) vocalizations and b) struggles, and the intensity of c) vocalization and d) struggles recorded during the backtest that was performed on 889 young and clinically healthy pigs at ∼27 days of age.

Figure 2 shows the distribution of backtest responses and Table 2 their summary statistics. Most pigs did not vocalize, and many pigs made few to no struggles during the 30 s backtest (Fig. 2a and b). Compared to the 60 s that is used for the backtest in some studies (Rohrer *et al*. 2013; Iversen *et al*. 2017), the 30 s we used could miscategorize pigs with higher latency to vocalize or struggle, although several studies have found inverse relationships between the latency of an animal to perform a behavior and the frequency of that behavior (D’Eath and Burn 2002; Zebunke *et al.* 2015). This implies that pigs that respond after 30 s in a longer test are expected to be identified as low responders in a 30 s test. The backtest was done at 27 days of age to allow for ∼5 days of acclimation while avoiding having to handle larger pigs. Because the backtest is instantaneous, we do not expect it to have an effect on stress hormone levels in hair collected at 40 days of age.

### Heritability

#### Stress hormone levels

Table 3 shows estimates of heritability for the evaluated traits. DHEA-S had the highest phenotypic variance of all hormones evaluated. Estimates of heritability were moderately high for the levels of cortisol and DHEA-S (0.33 and 0.31) and very low for cortisone (0.04) and DHEA-S (0.00). Several studies have reported similar heritability estimates for hair cortisol levels in Holstein cows (0.35 and 0.39 for winter and summer seasons, respectively, by Shi *et al*. 2021), and in vervet monkeys (0.31 by Fairbanks *et al*. 2011). Highly variable heritability estimates for cortisol have been reported for hair of human twins, ranging from 0 to 0.72 (Rietschel *et al.* 2017).

**Table 3.** Estimates of variance components, heritability, and the proportion of the phenotypic variance explained by litter effects for the natural log-transformed stress hormone levels and their ratios in hair of young pigs, and the backtest responses with associated standard errors (SE).

Ln (Hormones)	Heritability (SE)	Litter (SE)	Phenotypic variance
Cortisol	0.33 (0.10)	0.16 (0.05)	0.12 (0.01)
Cortisone	0.04 (0.07)	0.09 (0.04)	0.26 (0.01)
DHEA	0.00	0.11 (0.04)	0.11 (0.01)
DHEA-S	0.31 (0.10)	0.15 (0.05)	0.92 (0.01)
Cortisol + Cortisone (SOG)	0.10 (0.08)	0.15 (0.05)	0.15 (0.01)
DHEA + DHEA-S (SOD)	0.30 (0.10)	0.17 (0.05)	0.69 (0.04)
Ln (Hormone ratios)			
Cortisol/DHEA	0.23 (0.09)	0.12 (0.05)	0.15 (0.01)
Cortisol/Cortisone	0.00	0.00	0.19 (0.01)
Cortisone/DHEA	0.01 (0.05)	0.06 (0.04)	0.30 (0.02)
Cortisol/DHEA-S	0.38 (0.10)	0.15 (0.05)	0.76 (0.05)
Cortisone/DHEA-S	0.31 (0.10)	0.12 (0.05)	0.83 (0.05)
DHEA/DHEA-S	0.34 (0.09)	0.13 (0.05)	0.85 (0.05)
SOG/SOD	0.35 (0.10)	0.15 (0.05)	0.55 (0.03)
SOG/DHEA-S	0.37 (0.10)	0.14 (0.05)	0.76 (0.05)
Backtest			
Vocalization number	0.57 (0.08)	0.00	1.37 (0.10)
Struggles number	0.28 (0.08)	0.00	0.49 (0.03)
Vocalization intensity	0.26 (0.06)	0.04 (0.04)	4.69 (0.25)
Struggle intensity	0.29 (0.05)	0.00	4.61 (0.24)

Although DHEA is the active form of the hormone with antiglucocorticoid roles (Campbell 2020), we found its level in hair not to be heritable in these clinically healthy young pigs, while the level of DHEA-S was heritable (Table 3). Thus, although DHEA and DHEA-S tend to be used interchangeably in literature, these results provide evidence that DHEA and DHEA-S in hair of young and clinically healthy pigs may have a very different genetic basis, although the genetic correlation between them could not be estimated because of the low heritability of DHEA. Serum levels of DHEA-S have, however, been associated with healthier physiological profiles and general stress-buffering effects in humans (Maninger *et al.* 2009). To the best of our knowledge, no other study has estimated the heritability of DHEA or DHEA-S in hair but estimates for DHEA-S in human blood and saliva can range from low to as high as 0.65 after adjusting for the effect of age (Rotter *et al.* 1985; Goodarzi *et al.* 2015). The estimate of heritability of SOG (=cortisol + cortisone) was low (0.10), as it was dominated by the more variable cortisone level (Table 2), which had a low heritability estimate. In contrast, the estimate of heritability of SOD (=DHEA + DHEA-S) was similar to that for DHEA-S (0.30), as DHEA-S was much more variable than DHEA (Table 2).

All pigs evaluated in this study were barrows. There is varying information in literature about the effect of sex on the genetic basis of DHEA-S. For example, Rice *et al.* (1993) highlighted the significance of sex on heritability estimates for DHEA-S in human serum, with higher estimates for males than for females. In contrast, Nestler *et al.* (2002) reported no significant effect of sex on the additive genetic variance for DHEA-S in a human twin study, although there were significant phenotypic differences between the 2 sexes. Thus, our results may need to be reproduced in a mixed-sex population for robust application to gilts and sows. However, because samples were taken before sexual maturity, results may be similar for young females and intact males as observed here for young barrows.

#### Hormone ratios

Estimates of heritabilities of ratios of hormone levels are in Table 3. Genetic parameters for the ratios were not affected by the numerator/denominator order of the ratio since log-transformed values of the ratios were analyzed. Estimates of heritabilities for ratios were essentially zero for cortisol/cortisone and cortisone/DHEA and was 0.23 ± 0.09 for cortisol/DHEA. Heritability estimates for ratio's that included DHEA-S as one of its components ranged from 0.31 to 0.38, including SOG/SOD and SOG/DHEA-S, and they were higher than the estimates for the individual hormone levels included in the ratio. The ratio of cortisol/DHEA-S had the highest estimate, at 0.38 ± 0.10.

This is the first report on heritability estimates for ratios of stress hormones in hair of pigs. For stress hormones that are assumed to coregulate each other, their ratios may represent complex physiological interactions better than levels of the individual hormones (Sollberger and Ehlert 2016). For example, cortisol/cortisone reflects activity of the 11β-HSD isozymes type 1 and type 2 (Tomlinson and Stewart 2001). The cortisol/cortisone ratio was low (Table 2), suggesting that the 11β-HSD type 1 had a low activity during this phase. Although these pigs were maintained in temperature-controlled facilities, Escribano *et al.* (2023) also reported higher cortisone than cortisol levels in hair of heat-stressed crossbreds of Duroc Danbred × (Landrace × Large white) from 3 months of age when compared to those in cooled housing. Based on their results, Escribano *et al.* (2023) recommended cortisol/cortisone in hair as a metric for chronic stress produced by heat stress conditions in pigs. However, this ratio was not heritable in hair collected on these young and healthy pigs, although the heritability of cortisol was moderate (Table 3). This could indicate that in healthy young pigs, genetics contributes to the level of cortisol in circulation but its balance with cortisone is more environmentally modulated, probably through enzymatic activities of the 11-β HSD isozymes.

The cortisone/DHEA ratio can inform the balance between physiological stress response and anabolism in the animal, with a lower ratio indicating a more anabolic state with better stress resilience and tissue repair capabilities (van Uum *et al.* 2002; Teixeira *et al.* 2022). However, the heritability estimate for this ratio was very low (Table 3).

The ratios cortisol/DHEA-S and cortisone/DHEA-S had moderate heritability estimates (0.31 and 0.38) in hair of these clinically healthy young pigs (Table 3). Since serum levels of DHEA-S are associated with healthier physiological profiles and general stress-buffering effects (Maninger *et al.* 2009), pigs with a lower cortisol/DHEA-S or cortisone/DHEA-S ratio may have a higher physiological potential to tolerate chronic stress, possibly related to having higher reserves of DHEA-S that can easily be converted to DHEA to perform its antagonistic roles on glucocorticoids when the physiological need arises.

Although the biological significance of most of the ratios evaluated has not been explored, our results show that genetics contributes considerably to the variation of some of these ratios in hair of clinically healthy young pigs (Table 3), suggesting they can be modified by genetic selection. Overall, these results indicate that physiological response to noninfectious stressors can be captured by the levels of stress hormones in hair of clinically healthy young pigs and by their ratios, and that these traits exhibit considerable genetic variation.

#### Backtest responses

Heritability estimates for backtest responses ranged from 0.26 to 0.57, with VN having the highest estimate among the backtest responses evaluated (Table 3). Estimates of similar backtest response traits were within the same range in several studies (Bunter and Lansdowne 2004; Velie *et al.* 2009; Zebunke *et al.* 2015; Iversen *et al.* 2017) but were lower in Rohrer *et al*. (2013) and Scheffler *et al*. (2014).

### Litter effects

Estimates of litter effects ranged from 0.09 to 0.16 for hormone levels, from 0 to 0.15 for their ratios, and were essentially zero for backtest responses (Table 3). These results suggest that hormone levels in hair of healthy nursery pigs are significantly influenced by the common litter environment but only up to a degree. These results are supported by many neurobiological studies in mammals that have shown that the maternal environment can significantly modify an individual's HPA axis responses (Francis *et al.* 1999; Duthie and Reynolds 2013; de Kloet *et al*. 2014; Hennessy *et al*. 2015). Kazakou *et al*. (2023) showed that early life maternal care in animals and parental care in humans significantly impacts the mental health of offspring, as well as how they perceive situations as stressful, thereby affecting the physiological response to stressors.

Responses of pigs to the backtest were not significantly affected by the common litter environment (Table 3), which is not consistent with Bunter and Lansdowne (2004), who found moderate litter effect estimates associated with the number of escape attempts and the degree (intensity) of vocalizations, ranging from 10 to 34% of phenotypic variance. In addition, behavioral studies have reported that, artificially reared pigs significantly differ in behaviors from sow-reared pigs, such as having more aggression toward other pigs and more frequent and longer belly nosing (Rzezniczek *et al.* 2015). Schmitt *et al.* (2019) also showed that artificially reared pigs made more oral manipulations of conspecific tails and ears of littermates. These behaviors are induced when pigs are stressed by their social and physical environments, which points to the potential influence of the maternal environment on how pigs physically react to stressors. Although the above studies, in addition to our findings, indicate that the maternal environment plays a role in the response of pigs to stress, development of the physical stress responses of pigs may to a greater extent be modulated by the physical and social environment they interact with, including pen components, other littermates, and humans (Telkäranta and Edwards 2018). This is partly because pigs have evolved in a sensory rich environment, in which development is mostly driven by response to stimuli, received from the environment (Weller *et al.* 2019, 2020). Modern pig production, however, involves separating piglets from sows at an early age, which slows the development process of stress response traits, as they then tend to spend more time studying the environment (Schmitt *et al.* 2019).

### Correlations among hormone traits

Table 4 shows estimates of phenotypic and genetic correlations between hormone traits, including ratios of hormone levels that were more highly heritable. Genetic correlation estimates (${\hat{r}}_{g}$) for pairs involving DHEA did not converge because it was not heritable. Phenotypic correlation estimates (${\hat{r}}_{p}$) among the levels of individual hormones were all moderately high and positive, ranging from 0.26 to 0.65.

**Table 4.** Estimates of genetic (below the diagonal) and phenotypic (above the diagonal) correlations among the levels of stress hormones in hair of young healthy pigs and their responses to the backtest.

	Cortisol	Cortisone	DHEA	DHEA-S	SOG	SOD	Cortisol/cortisone	Cortisol/DHEA-S	Cortisone/DHEA-S	SOG/SOD	SOG/DHEA-S	Vocalization number	Struggles number	Vocalization intensity	Struggle intensity
Cortisol		0.55 (0.03)	0.58 (0.06)	0.45 (0.03)	0.73 (0.02)	0.49 (0.03)	0.21 (0.04)	−0.05 (0.04)	−0.14 (0.04)	−0.13 (0.04)	−0.15 (0.04)	−0.03 (0.04)	0.08 (0.04)	0.02 (0.05)	−0.01 (0.04)
Cortisone	0.99 (0.12)*^a,d^*		0.26 (0.08)	0.37 (0.03)	0.95 (0.00)	0.38 (0.03)	−0.78 (0.02)	−0.19 (0.04)	0.19 (0.04)	0.07 (0.04)	0.03 (0.04)	0.00 (0.00)	0.01 (0.04)	0.02 (0.04)	0.00 (0.04)
DHEA	nc	nc		0.65 (0.07)	0.28 (0.04)	0.43 (0.05)	0.04 (0.04)	−0.17 (0.04)	−0.17 (0.04)	−0.31 (0.05)	−0.28 (0.05)	0.00 (0.00)	0.03 (0.04)	0.03 (0.04)	0.00 (0.04)
DHEA-S	0.21 (0.23)	0.78 (0.86)	nc		0.42 (0.03)	0.98 (0.00)	−0.07 (0.04)	−0.94 (0.01)	−0.83 (0.01)	−0.90 (0.01)	−0.92 (0.01)	0.06 (0.04)	0.10 (0.04)	0.04 (0.04)	0.04 (0.05)
SOG*^e^*	nc	nc	nc	0.36 (0.35)		0.46 (0.03)	−0.68 (0.04)	−0.17 (0.04)	0.08 (0.04)	0.01 (0.04)	−0.02 (0.04)	−0.04 (0.04)	−0.03 (0.04)	−0.03 (0.04)	−0.01 (0.04)
SOD*^f^*	0.27 (0.24)	0.80 (0.60)*^c,d^*	nc	1.00 (0.00)*^a^*	0.43 (0.36)		−0.07 (0.05)	−0.91 (0.01)	−0.83 (0.01)	−0.89 (0.01)	−0.89 (0.01)	0.03 (0.05)	−0.02 (0.04)	0.04 (0.04)	−0.03 (0.04)
Cortisol/cortisone	0.74 (0.98)	0.25 (1.28)	nc	−0.93 (2.09)	nc	nc		0.17 (0.04)	−0.43 (0.04)	−0.31 (0.05)	−0.25 (0.05)	0.03 (0.05)	0.00 (0.05)	0.00 (0.04)	−0.01 (0.04)
Cortisol/DHEA-S	0.18 (0.22)	−0.26 (0.26)	nc	−0.93 (0.02)*^a^*	0.13 (0.40)	−0.93 (0.04)*^a^*	0.99 (1.29)		0.01 (0.04)	0.93 (0.01)	1.00 (0.01)	−0.02 (0.04)	0.22 (0.11)	0.02 (0.04)	0.07 (0.04)
Cortisone/DHEA-S	0.05 (0.25)	−0.53 (0.75)	nc	−0.99 (0.03)*^a^*	−0.35 (0.49)	−0.98 (0.05)*^a,d^*	0.66 (1.10)	−0.12 (0.18)		0.97 (0.00)	0.98 (0.00)	0.00 (0.04)	0.07 (0.05)	0.04 (0.05)	0.08 (0.05)
SOG/SOD	0.15 (0.24)	−0.60 (0.89)	nc	−0.99 (0.02)*^a,d^*	−0.24 (0.41)	−0.98 (0.03)*^a,d^*	nc	nc	nc		0.98 (0.00)	−0.07 (0.04)	0.01 (0.04)	−0.06 (0.04)	0.03 (0.04)
SOG/DHEA-S	0.20 (0.32)	−0.54 (0.81)	nc	−0.98 (0.03)*^a,d^*	−0.18 (0.37)	−0.96 (0.04)*^a,d^*	nc	nc	nc	nc		−0.07 (0.04)	0.00 (0.04)	−0.06 (0.04)	0.02 (0.04)
Vocalization number	0.24 (0.19)*^c^*	0.20 (0.41)	nc	0.01 (0.18)	0.26 (0.39)	0.04 (0.14)	nc	0.07 (0.16)	−0.01 (0.19)	−0.02 (0.17)	−0.01 (0.17)		0.56 (0.03)	0.85 (0.01)	0.54 (0.03)
Struggles number	0.15 (0.25)	0.51 (0.75)	nc	0.04 (0.25)	0.35 (0.55)	−0.03 (0.24)	nc	0.04 (0.22)	0.04 (0.26)	0.05 (0.22)	0.07 (0.22)	0.64 (0.13)*^a^*		0.54 (0.03)	0.75 (0.02)
Vocalization intensity	0.50 (0.24)*^b^*	0.27 (0.29)	nc	−0.10 (0.16)	0.52 (0.55)	0.01 (0.24)	nc	0.16 (0.20)	0.17 (0.24)	0.17 (0.22)	0.16 (0.21)	0.98 (0.03)*^a,d^*	0.85 (0.17)*^a^*		0.53 (0.03)
Struggle intensity	0.06 (0.24)	0.12 (0.31)	nc	0.01 (0.24)	0.23 (0.46)	−0.16 (0.23)	nc	0.01 (0.22)	0.03 (0.26)	0.12 (0.23)	0.11 (0.23)	0.60 (0.13)*^a^*	0.99 (0.09)*^a,^*^d^	0.72 (0.13)*^a^*	

“nc” indicates no convergence.

Significance level of being different from zero: ^a^ *P* < 0.001. ^b^ *P* ≤ 0.15. ^c^ *P* ≤ 0.25.

^*d*^Estimate is not significantly different from 1 or −1 at *P* ≤ 0.05.

^*e*^Sum of glucocorticoids (SOG) = cortisol + cortisone.

^*f*^Sum of DHEA(S) (SOD) = DHEA + DHEA-S.

Estimates of genetic correlations among the levels of individual hormones were positive and ranged from low to very high (0.12 to 0.96), with cortisone showing a very high ${\hat{r}}_{g}$ with cortisol (0.99 ± 0.12) and was not significantly different from one (*P* ≤ 0.05). This indicates that the levels of cortisone and cortisol in hair of these clinically healthy young pigs could have a very similar genetic basis, likely because they have similar biosynthetic pathways. Their levels in hair may, however, differ as a result of differential activity of the isozymes of 11β-HSD during this early phase of the pig's life. Cortisone also had a high ${\hat{r}}_{g}$ with DHEA-S (0.78) but with a high standard error and not significantly different from zero because of the low heritability of cortisone. The ${\hat{r}}_{g}$ between cortisol and DHEA-S was also low and not significantly different from zero.

The ${\hat{r}}_{g}$ between hormone ratios were poorly estimated and did not converge or were not significantly different from zero (Table 4). Genetic correlation estimates of hormone levels with their ratios generally had high standard errors, but since there is no literature to compare them to, they could not be validated. Ratios that included DHEA-S as one of its components (including through SOD) had very large ${\hat{r}}_{g}$ with both DHEA-S and SOD (absolute value ≥0.93 and not significantly different from 1), suggesting that most of the genetic variation in these ratios was driven by DHEA-S, although their phenotypic correlations were close to zero.

Overall, the moderate phenotypic and genetic correlation estimates between hormone levels, and the moderate heritabilities estimated for some, indicate that they share some genetic factors and operate together in the interactive physiological environment during stress response. These results agree with the published modes of action of the evaluated hormones. That is, DHEA(S) are known to have antagonistic roles on the effects of glucocorticoids during stress response (Kimonides *et al.* 1999; Guilliams and Edwards 2010) but participate in restoring physiological homeostasis. During stress response, cortisol levels increase, followed by an increase in DHEA through increased production from the adrenal gland (Dutheil *et al*. 2021), and production of DHEA-S. This “leader-follower” interactive mode of action by these 2 groups of hormones is what is captured by their moderately high positive phenotypic correlations (Table 4). The interaction between these 2 groups of hormones can also be regulatory, since DHEA has been shown to block translocation of the glucocorticoid receptor to the nucleus in in-vitro studies (Maninger *et al.* 2009).

### Correlations among backtest traits

The 4 backtest responses generally had high estimates of phenotypic (0.53 to 0.85) and genetic correlations (0.60 to 0.99) among themselves (Table 4). Genetic correlation estimates were, in particular, high and not significantly different from one between SI and SN (0.99 ± 0.09) and between VI and VN (0.98 0.03). These pairs of responses also had strong phenotypic correlation estimates, indicating that pigs that made many struggles during the test also tended to score high on the intensity score, and those that made many vocalizations were also scored to have vocalized the loudest. These findings suggest that it is sufficient to record either the intensity or the number for these traits. Based on its higher heritability, VN may be the most representative response of those analyzed. Scheffler *et al*. (2014) proposed that either the latency to vocalize (time until the first vocalization of a pig during the backtest) or the number of vocalizations should be recorded but not both. They made a similar recommendation for the latency to struggle and the number of struggles. Several studies have proposed the use of struggles as the sole recorded response in the backtest (Ruis *et al.* 2000; Geverink *et al.* 2004). Iversen *et al.* (2017), however, noted that the latency of pigs to vocalize was shorter than latency to struggle, suggesting that a 30-second duration for the backtest could cause late responders to be recorded as nonresponders for struggles. However, studies have reported inverse relationships between the latency to perform a behavior and the frequency of that behavior (D’Eath and Burn 2002; Zebunke *et al.* 2015), hence a longer experimental duration is not required when struggle traits (e.g. SN and SI) are recorded as the sole backtest response phenotypes. Overall, the decision of which response to record during a backtest will depend on what the data will be used for, labor availability, heritability of the response, and its genetic correlations with the target traits in the breeding program, among others.

### Correlations between hormone and backtest traits

The backtest is a standard measure of the coping response style of pigs to this uncomfortable noninfectious stressor. Thus, we used it to validate the potential of stress hormone levels in hair to inform on the innate response style of pigs to noninfectious stressors such as weaning, transportation, mixing, castration, and others. Although the backtest was performed about 2 weeks before the hair was collected, the coping style it assesses reflects a coherent set of behavioral and physiological stress responses that is consistent over time (Koolhaas *et al.* 1999), suggesting it can be performed at any time. In addition, because stress caused by the backtest is acute, it is not expected to have a large effect on stress hormones in the sampled hair, which captures stress hormones deposited in hair during the entire time that the hair was grown.

Estimates of phenotypic correlations between hormone and backtest traits were generally low (−0.03 to 0.22) (Table 4). Other studies have also shown nonconvincing phenotypic correlations between HPA axis activity and coping style, such as in rats bred for aggression (Koolhaas *et al.* 2007), while Veenema *et al.* (2003)found that their correlation depended on the type and duration of the stressor used. Van Reenen *et al.* (2005), using a principal component analysis in cattle that were faced with a variety of challenging situations, also showed that parameters of the HPA axis loaded significantly on the emotionality factor but not on the coping style factor. Van Erp-van der Kooij *et al.* (2003) also observed no phenotypic correlation between saliva cortisol levels in pigs and the number of escape attempts during the backtest (which corresponds to SN in our study). Therefore, the low phenotypic correlations of backtest responses with hair hormone levels observed in our study can be attributed to the difference in the nature of the responses that are reflected by stress hormone levels in hair and backtest responses.

Estimates of genetic correlations of backtest responses were, however, moderately high and positive with the levels of both glucocorticoids (cortisol and cortisone) but very low with DHEA-S (Table 4). Most of these estimates were, however, not significantly different from zero at *P* ≤ 0.05. Among the significant estimates, cortisol had moderate positive genetic correlation estimates with both VN (0.24 ± 0.19) and VI (0.50 ± 0.24) but not with the struggle traits. This could be because pigs are a highly social species that will vocalize strongly when in danger or under threat. Vocalizing is perceived by pigs as a call to other members of the family to defend the threatened individual (Støier *et al.* 2016). Genetic correlation estimates were essentially zero between backtest responses and hormone ratios.

Overall, based on the estimates of genetic correlations and heritabilities, we can conclude that the level of cortisol in hair of clinically healthy nursery pigs is a potential genetic indicator of the pig's coping style of response to noninfectious stressors. This aligns with reports by Martin *et al*. (2011), who suggested that in vertebrates, coping with stressors begins with cortisol in fish and many mammals or with corticosterone in amphibians, reptiles, and birds.

### Genome-wide association studies

#### QTL identified

**Fig. 3.**
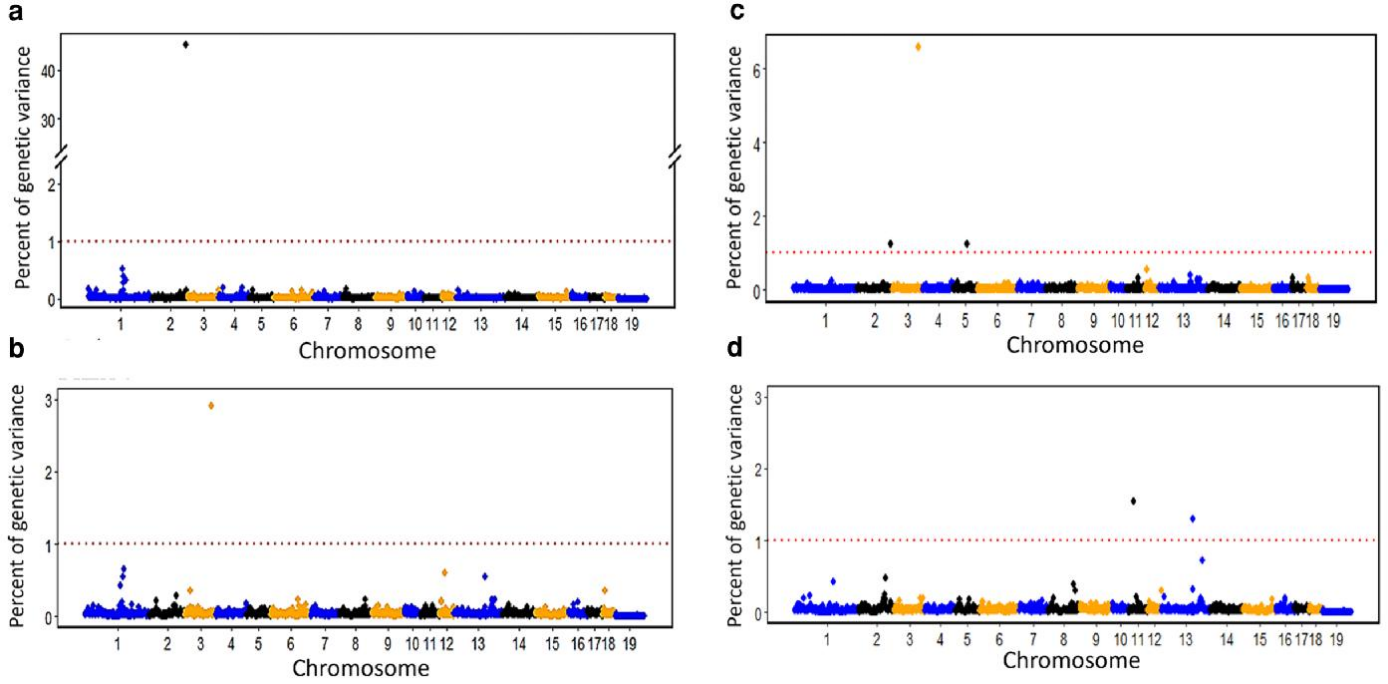
Univariate GWAS results: Manhattan plots for the percentage of genetic variance explained by nonoverlapping 1 Mb windows for the natural log-transformed levels of a) cortisol, b) DHEA-S, c) cortisol/DHEA-S, and d) cortisone/DHEA-S in hair of young and clinically healthy pigs. Windows that explained more than 1% genetic variance (above the threshold line) were considered QTL.

This study presents the first GWAS for levels of cortisol, cortisone, DHEA, and DHEA-S and their ratios in hair of pigs. Significant QTL were defined as 1-Mb window that explained ≥1% of EGV. Manhattan plots in Fig. 3 show a major QTL at 144 Mb on SSC chromosome 2 for cortisol and cortisol/DHEA-S, explaining 45.3 and 1.2% of EGV, respectively, as well as other QTL with relatively smaller estimated effects for DHEA-S, cortisol/DHEA-S, and cortisone/DHEA-S (see Table 5). No QTL were identified for cortisone, DHEA, and cortisol/cortisone (Supplementary Fig. 2). This study also reports the first GWAS results for backtest responses (see Supplementary Fig. 3), and QTL identified for VN and VI in Table 5. No QTL were identified for SN and SI at the 1% EGV threshold, but a region on SSC4 was declared suggestively important based on functional relevance of the genes located in this region to these 2 traits and explained 0.5% of the EGV for each of these traits (Table 5).

**Table 5.** QTL identified for the levels of stress hormones in hair of young healthy pigs and for their ratios, and the estimated percentage of genetic variance they explained, significant SNPs with a PIP ≥ 1%, and candidate genes identified in 5 Mb regions centered on the 1 Mb QTL windows.

SSC	1 Mb window	Trait	% genetic variance	Significant SNPs (PIP)	Candidate genes
1*^a^*	147	Cortisol	0.5	rs341220241 (0.02)	*CYB5A*
	164	DHEA-S	0.6	rs346150735 (0.02)	*SMAD6*
2	144	Cortisol	45.3	rs330467924 (1.00) rs336612332 (1.00)	*NR3C1, HDAC3, SRA1*
		Cortisol/DHEA-S	1.2	rs320551572 (0.07) rs81333702 (0.05)	
3	114	DHEA-S	2.9	rs320869146 (0.10) rs81330468 (0.09)	*FKBP1B, NCOA1*
		Cortisol/DHEA-S	6.6	rs320869146 (0.26) rs345029282 (0.24)	
4*^a^*	97	Struggle intensity	0.5	rs344708496 (0.03)	*TMOD4, SCNM1, S100A10, S100A11*
		Struggles number	0.5		
5	59	Cortisol/DHEA-S	1.2	rs80953780 (0.06)	*EMP1, ETV6, ENSSSCG00000056452*
9	89	Vocalization number	11.1	rs330516450 (0.02) rs337375072 (0.01)	*ITGB8, HDAC9, ABCB5*
11	19	Cortisone/DHEA-S	1.5	rs327560275 (0.05)	*NUDT15*
	76	Vocalization intensity	1.6	rs342205313 (0.06)	*COL4A1, COL4A2*
12	2	Vocalization intensity	1.7	rs331493124 (0.16)	*SLC16A3*
13	135	Cortisone/DHEA-S	1.3	rs328427261 (0.02)	*OSBPL11*
16	63	Vocalization intensity	1.0	rs81461064 (0.06)	*ADRA1B*
18	24	Vocalization number	1.3	rs335652397 (0.02) rs321278790 (0.01)	*CADPS2, KCND2*

^*a*^A region that explained <1% genetic variance.

Table 5 also lists the lead SNPs from the 650 K panel that had posterior inclusion probabilities (PIP) (i.e. the proportion of iterations of the chain that the SNP was included in the model) ≥ 0.01. The major QTL for cortisol on SSC2 had 2 lead SNPs with PIP = 1.00, while the QTL for cortisol/DHEA-S had 2 lead SNPs with PIP of 0.24 and 0.26. The PIP for the lead SNPs for all other QTL was <0.1.

#### Candidate genes in QTL regions

Candidate genes were identified in 5-Mb regions centered around each identified 1-Mb QTL window and are listed in Table 5. For the backtest responses, QTL identified for VN on SSC9 and18 harbored the *ITGB8, HDAC9, ABCB5, CADPS2*, and *KCND2* genes. The *ITGB8* gene is involved in cell-to-cell and cell-to-extracellular matrix interactions and hence may facilitate movement of muscle as 1 unit during vocalizing. The *ABCB5* gene is involved in cellular transportation of polyamines, which are involved in protein synthesis, mitochondrial function, and energy production (Bekebrede *et al.* 2020). The *KCND2* gene is involved in excitability of smooth muscle cells as a voltage-gated potassium channel (Xiaobo *et al.* 2021), while the *HDAC9* gene is reported to be involved in suppressing stress signals, which may be necessary for regulating the input signals that excite muscle during muscle contraction to avoid over-excitation. This gene has also been shown to suppress cardiac hypertrophy, which is the main process through which an adult heart responds to stress signals (Chang *et al.* 2004). The *CADPS2* gene is 1 of the genes responsible for the uptake, storage, and release of neurotransmitters (Subramanian *et al.* 2005).

The QTL for VI on SSC11, 12, and 16 (Table 5) harbored the *COL4A1, COL4A2, SLC16A3*, and *ADRA1B* genes. COL4A1 and COL4A2 are basement membrane proteins that participate at the attachment points of muscles, which affect overall stability of the skeletal system during movements and, hence, provide a supportive framework that facilitates muscle contraction and movement. Mutations in the *COL4A1* gene have been shown to result in myopathy in mice, characterized by muscle weakness and split muscle fibers (Kuo *et al.* 2012). The *SLC16A3* gene has been associated with pH regulation and energy metabolism in muscles by transporting L-lactate across plasma membranes (Juel and Halestrap 1999). This gene is strongly overexpressed in highly glycolytic and anaerobic tissues (Bosshart *et al.* 2019) and thus may modulate the intensity of muscle movements during intense vocalization. Other members of the SLC family of genes (*SLC18A2* and *SLC25A16*) have been associated with temperament in cattle (Kolbehdari *et al*. 2008; Garza-Brenner *et al.* 2017; Macleod *et al*. 2019). The *ADRA1B* gene produces the alpha-1B adrenergic receptor which, together with the 5-HT2A receptor, has been shown to entirely regulate dopamine release (Auclair *et al*. 2004).

Although no QTL were identified for SI and SN (Supplementary Fig. 3), a region on SSC4 that explained 0.5% EGV for SI and for SN was declared suggestively important (Table 5), as it harbors several genes of interest, including the *TMOD4*, *SCNM1,* and several calcium-binding protein genes, including *S100A10* and *S100A11*. The TMOD4 protein regulates filament elongation and depolarization when working with tropomyosin in muscles (Almenar-Queralt *et al*. 1999; Mazelet *et al*. 2016). The direct role of the *SCNMI* gene product in muscle movement has not been well established but has been shown to influence the severity and progression of dystocia in mice and humans (Sprunger *et al*. 1999; Buchner *et al*. 2003; Howell *et al*. 2007), a condition characterized by involuntary muscle contractions. Lastly, the *S100* gene family plays a role in calcium ion movements during muscle contraction and/or emotional responses, which may vary in intensity based on the stress faced (McCarty 2020).

For stress hormones, different studies have reported QTL for plasma levels of DHEA in chickens (Fallahsharoudi *et al*. 2017) and for serum levels of DHEA-S in humans (Zhai *et al*. 2011; Ruth *et al*. 2016; Pott *et al*. 2019; Miranda *et al*. 2022), but no study has reported associated genomic regions for levels of DHEA or DHEA-S in hair for any organism. The QTL on SSC3 for both DHEA-S and cortisol/DHEA-S (Table 5) harbored the *FKBP1B* and *NCOA1* genes. The protein encoded by the *FKBP1B* gene is a member of the immunophilin family and has been reported to be involved in counteracting the immunosuppressive and toxic effects of FK506 and rapamycin in humans, thereby reinstating Ca^2+^-dependent transcription of lymphokine genes in T-cells (Dumont *et al*. 1992). It should be noted that immunophilins are directly involved in activation and nuclear localization of the GR (Grad *et al*. 2007). Therefore, this gene could be involved in the antagonistic functions of DHEA(S) on cortisol during stress response by facilitating nuclear translocation of the GR to repress target stress-responsive genes. The protein encoded by the *NCOA1* gene acts as a transcriptional coactivator for steroid and nuclear hormone receptors (Chen and Li, 1998; Chen *et al*. 2024), thereby enhancing transcription of target genes that are responsive to steroid hormones, which could affect biosynthesis of DHEA(S) and other steroid hormones.

The QTL on SSC5 was unique for cortisol/DHEA-S and harbors the *EMP1, ETV6*, and *ENSSSCG00000056452* genes (Table 5). The *EMP1* gene encodes the epithelial membrane protein 1, for which a direct link to either cortisol or DHEA-S has not been published but it was found to be overexpressed in poor responders to prednisolone in humans (Singh *et al*. 2021). Similarly, there no clear link has been reported to date between the *ETV6* gene and cortisol, DHEA-S, or their ratio, but ETV6 has been identified as a transcriptional repressor for inflammatory genes during stress hematopoiesis in mice (Bloom *et al*. 2023). The *ENSSSCG00000056452* gene has not been annotated to date, but it harbors the lead SNPs for this QTL.

Candidate genes for the QTL on SSC11 and 13 for cortisone/DHEA-S include the *NUDT15* and *OSBPL11* genes, respectively (Table 5). The *OSBPL11* gene encodes a member of the oxysterol-binding protein (OSBP) family with a highly conserved C-terminal OSBP-like sterol binding domain. OSBPs bind oxysterols, which function as intermediates in the synthesis of steroid hormones (Björkhem and Diczfalusy 2002; Olkkonen 2009; Olkkonen *et al*. 2012). Oxysterols could, therefore, be involved in modification of the cortisone/DHEA-S ratio by participating in membrane signaling, lipid metabolism, and sterol transport in the cells (Weber-Boyvat *et al*. 2013).

Although not significant based on the GWAS, 2 regions of interest were identified on SSC1 for cortisol and DHEA-S (0.5 and 0.6% EGV), respectively, that could be biologically relevant (Fig. 3). The region at 147 Mb for cortisol harbors the *CYB5A* gene, which plays a role in the biosynthesis of cortisol and DHEA(S) (Storbeck *et al*. 2013; Auchus and Miller 2015; Schiffer *et al*. 2019). The region at 164 Mb for DHEA-S harbors the *SMAD6* gene, which is involved in transforming TGF-β1 signaling (Derynck and Zhang 2003) and, hence, may be involved in pathophysiological response to stress.

For the major QTL for cortisol on SSC5, candidate genes include the *NR3C1*, *SRA1*, and *HDAC3* genes (Table 5). The *NR3C1*gene encodes the glucocorticoid receptor (GR) to which cortisol and other glucocorticoids bind. The GR then translocates to the nucleus to regulate transcription or repression of target stress-responsive genes. Genetic variants in the *NR3C1* gene have previously been associated with plasma cortisol levels in pigs (Muráni *et al*. 2012; Reyer *et al*. 2016; Terenina *et al.* 2025), in cattle (Poleti *et al*. 2015), and in humans (Plieger *et al*. 2018), as well as in human hair (Nordkap *et al*. 2022). The *HDAC3* gene is involved in regulation of several oxidative stress-related processes and molecules via its deacetylase and nonenzymatic activities (He *et al*. 2023). This is important because Flaherty *et al*. (2017) showed that noninfectious stressors can increase cortisol production in pigs, which can induce oxidative stress. In addition, stress response in animals involves post-translational modification of histone proteins to alter gene expression (Nunez-Vazquez *et al*. 2022). Therefore, the *HDAC3* gene product could influence the expression of glucocorticoid responsive elements, thereby regulating activity of the HPA axis. The *SRA1* gene encodes a long noncoding RNA (lncRNA) that acts as a coactivator of steroid receptors, including the GR, to modulate the transcription of various genes by coordinating the functions of various transcription factors and by enhancing steroid receptor-dependent gene expression (Liu *et al*. 2016).

#### Fine mapping the major QTL for cortisol

**Fig. 4.**
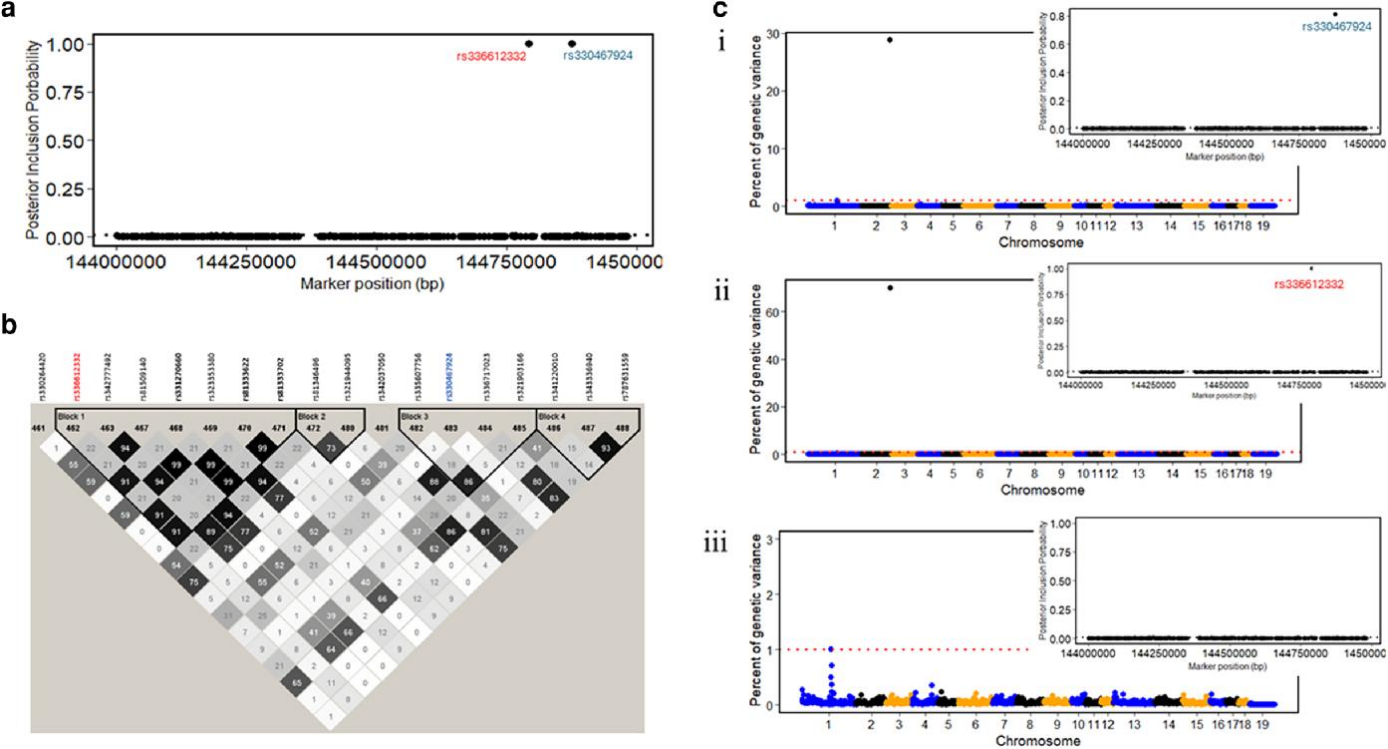
Fine mapping: lead SNPs in the major QTL for cortisol and their effect on the QTL when fitted individually or simultaneously as covariates. a) Scatter showing the PIP of lead SNPs in the 1 Mb major QTL at 144 Mb on SSC2 for cortisol levels in hair of healthy nursery pigs from a GWAS based on the 650 K SNP panel and b) the pair-wise LD (*r*^2^) between the SNPs neighboring them. c) Manhattan plots show the percent genetic variance explained by the major QTL when genotypes of the lead SNPs were fitted in the GWAS model individually (i and ii) and simultaneously (iii) as covariates.

The GWAS using the 650 K SNP genotypes identified 2 lead SNPs with a PIP of 1.00 in the 1-Mb QTL window at 144 Mb on SSC2 for cortisol (rs336612332 and rs330467924) while other SNPs in the region had near-zero PIP (Fig. 4a). These 2 SNPs, therefore, captured most of the effect of this QTL but were surprisingly not in high LD with each other (Fig. 4b). To further investigate these associations, the genotypes at these 2 lead SNPs (i.e. rs336612332 and rs330467924) were fitted as fixed covariates in the univariate form of the GWAS model, either individually or simultaneously. Results in Fig. 4c show that fitting either 1 of the 2 SNPs (rs336612332 or rs330467924) as a covariate in the GWAS model did not fully capture the effect of this QTL. However, fitting both simultaneously completely explained the QTL effect (Fig. 4c (iii)). Given the low LD between these 2 SNPs, this suggests that they either each captured part of the effect of the same QTL or that the region has 2 QTL and each of the lead SNPs captured the effect of 1 of the QTL.

For deeper fine mapping, a GWAS was performed with SNP genotypes in this 1-Mb QTL that were imputed to WGS. Results showed that 2 of the WGS-imputed SNPs had larger PIP, i.e. rs341258564 with PIP = 0.8 and rs335962816 with PIP = 0.2 (Supplementary Fig. 7a), while all other SNPs, including the original 2 lead SNPs from the 650 K genotypes identified in Table 5, had PIP <0.01. The 2 WGS-imputed lead SNPs were in very high LD with each other (*r*^2^ = 0.97) (Supplementary Fig. 7b). The WGS-imputed SNP with the highest PIP (rs341258564) was in moderate LD (*r*^2^ = 0.55) with 1 of the original 650 K lead SNPs (rs336612332) but in very low LD (*r*^2^ = 0.01) with the other 650 K lead SNP (rs330467924) (Supplementary Fig. 7b). However, fitting genotypes for this WGS-imputed lead SNP as a covariate in the GWAS model explained most of the QTL effect for cortisol in this region (Supplementary Fig. 7c), reducing the estimate of the genetic variance based on the random SNP effects from 0.039 to 0.019 and its heritability estimate from 0.33 to 0.20.

Among the 863 pigs with data on cortisol levels, 19.5% of the pigs were heterozygous for the WGS-imputed lead SNP, 80% were homozygous for the allele that increased cortisol levels, and 0.5% were homozygous for the minor allele. Phased WGS genotypes for the 1 Mb region around the WGS-imputed lead SNP identified 2 haplotypes that carried the minor allele at WGS-imputed lead SNP, of which one was present among Landrace haplotypes in the reference panel established by Ding *et al.* (2023) and the other among Yorkshire haplotypes. Frequencies of these Landrace and Yorkshire haplotypes among the F1 pigs were 6.0 and 4.1%, respectively, suggesting that frequencies of the allele at the WGS-lead SNP that reduces cortisol levels were 12.0 and 8.2% in the Landrace and Yorkshire parental lines.

The estimate of the substitution effect for the minor allele at the WGS-imputed lead SNP was −0.35 ± 0.03 on the natural log scale, which corresponds to a significant reduction in hair cortisol levels (*P* < 0.001) by 30 ± 6% on the original scale for an extra copy of the minor allele (mean genotypic values: 11.9 pg/mg for heterozygotes and 8.4 pg/mg for homozygotes of the minor allele). Fitting separate covariates depending on breed origin of the minor allele resulted in estimates of −0.39 ± 0.04 on the log scale for Landrace haplotypes and −0.30 ± 0.04 for Yorkshire haplotypes, with the difference approaching significance (*P* = 0.11). Thus, the causative variant for this QTL that reduces cortisol was present at a low frequency in both Landrace and Yorkshire parental lines and may have a larger effect for Landrace than for Yorkshire. Genotyping of the parental lines for the WGS-lead SNP and estimation of its effects on hair cortisol levels in purebreds is required to confirm these results.

Table 6 details estimated effects of an extra copy of the minor allele at the WGS-imputed lead SNP on all hormone and backtest traits evaluated, expressed in phenotypic standard deviation units. We did not observe a significant interaction between SNP genotype and company at *P* ≤ 0.05, suggesting that these results applied to pigs from all 7 companies. The allele associated with an increase in cortisol levels was, however, fixed for 1 of the companies. Worth noting was that an extra copy of the minor allele at this SNP also reduced cortisone levels by 19 ± 4%. Fitting the genotype of this WGS-imputed lead SNP as a fixed covariate for both cortisol and cortisone in the bivariate model did not affect the ${\hat{r}}_{g}$ between them. This indicates that the high ${\hat{r}}_{g}$ between levels of cortisol and cortisone (0.99, see Table 4) is equally driven by this QTL and by regions of the genome beyond this major QTL. Overall, the WGS-imputed lead SNP had significant effects on levels of glucocorticoids (and their ratios) in hair of nursery pigs but showed no significant effects on levels of DHEA, DHEA-S, or DHEA/DHEA-S. Among backtest responses, an extra copy of the minor allele at the WGS-imputed lead SNP significantly increased VN (*P* ≤ 0.05), by 5 ± 2%, but had no significant effects on the other backtest responses.

**Table 6.** Estimates of the minor allele substitution effect at SNP rs341258564 for all traits analyzed, expressed in phenotypic standard deviation units, and the variability of the estimates across all companies.

				SNP effect by company					
ln (Hormone traits)	Main effect	*P*-value	h^2^-adj*^a^* (SE)	A	C	D	E	F	*P*-value
Cortisol	−1.12	<0.01	0.19 (0.09)	−1.03	−0.79	−0.98	−1.07	−1.28	0.85
Cortisone	−0.39	<0.01	0.07 (0.07)	−0.25	−0.44	−0.42	−0.42	−0.70	0.73
DHEA	0.09	0.37	0.00	−0.11	−0.20	0.19	0.19	0.31	0.54
DHEAS	0.02	0.84	0.33 (0.09)	−0.18	0.34	0.00	0.02	0.05	0.57
Cortisol + Cortisone (SOG)	−0.67	<0.01	0.08 (0.08)	−0.53	−0.59	−0.72	−0.74	−0.90	0.84
DHEA + DHEA-S (SOD)	0.01	0.85	0.30 (0.10)	−0.19	0.26	0.01	0.04	0.00	0.69
Cortisol/Cortisone	−0.34	<0.01	0.00	−0.83	−0.53	−1.10	−1.10	−1.41	0.73
Cortisol/DHEA	−0.99	<0.01	0.17 (0.08)	−0.53	−0.14	−0.30	−0.39	−0.21	0.10
Cortisol/DHEAS	−0.43	<0.01	0.33 (0.09)	−0.19	−0.68	−0.39	−0.48	−0.59	0.53
Cortisone/DHEA	−0.43	<0.01	0.0 (0.06)	0.00	0.00	0.00	0.00	0.00	0.32
Cortisone/DHEAS	−0.25	0.01	0.32 (0.10)	0.04	−0.58	−0.22	−0.28	−0.49	0.35
DHEA/DHEAS	0.01	0.91	0.35 (0.09)	0.15	−0.39	0.08	0.02	0.10	0.43
SOG/SOD	−0.39	<0.01	0.32 (0.10)	−0.09	−0.58	−0.40	−0.45	−0.48	0.53
SOG/DHEA-S	−0.35	<0.01	0.35 (0.10)	−0.06	−0.62	−0.31	−0.39	−0.48	0.44
Backtest responses									
Vocalization number	0.25	0.01	0.58 (0.07)	0.99	0.05	0.25	0.26	0.28	0.88
Vocalization intensity	−0.09	0.33	0.26 (0.06)	0.02	0.04	−0.19	−0.02	0.00	0.90
Struggles number	0.06	0.57	0.29 (0.08)	0.89	−0.14	0.32	−0.28	0.11	0.15
Struggles intensity	−0.03	0.73	0.29 (0.05)	0.18	0.08	−0.13	0.04	0.08	0.92

^*a*^Heritability of the trait when the WGS-imputed lead SNP was fitted as a fixed covariate.

It is worth noting that this major QTL for cortisol was also found to be associated with VN (Fig. 6c) but not with VI. Adjusting for the WGS-imputed lead SNP reduced the ${\hat{r}}_{g}$ between cortisol and VN from 0.24 ± 0.19 to 0.18 ± 0.22. This suggests that a considerable portion of the covariance between cortisol and VN is driven by the major QTL. However, adjusting for the WGS-imputed lead SNP increased the ${\hat{r}}_{g}$ between cortisol and VI from 0.50 ± 0.24 to 0.55 ± 0.34.

**Fig. 5.**
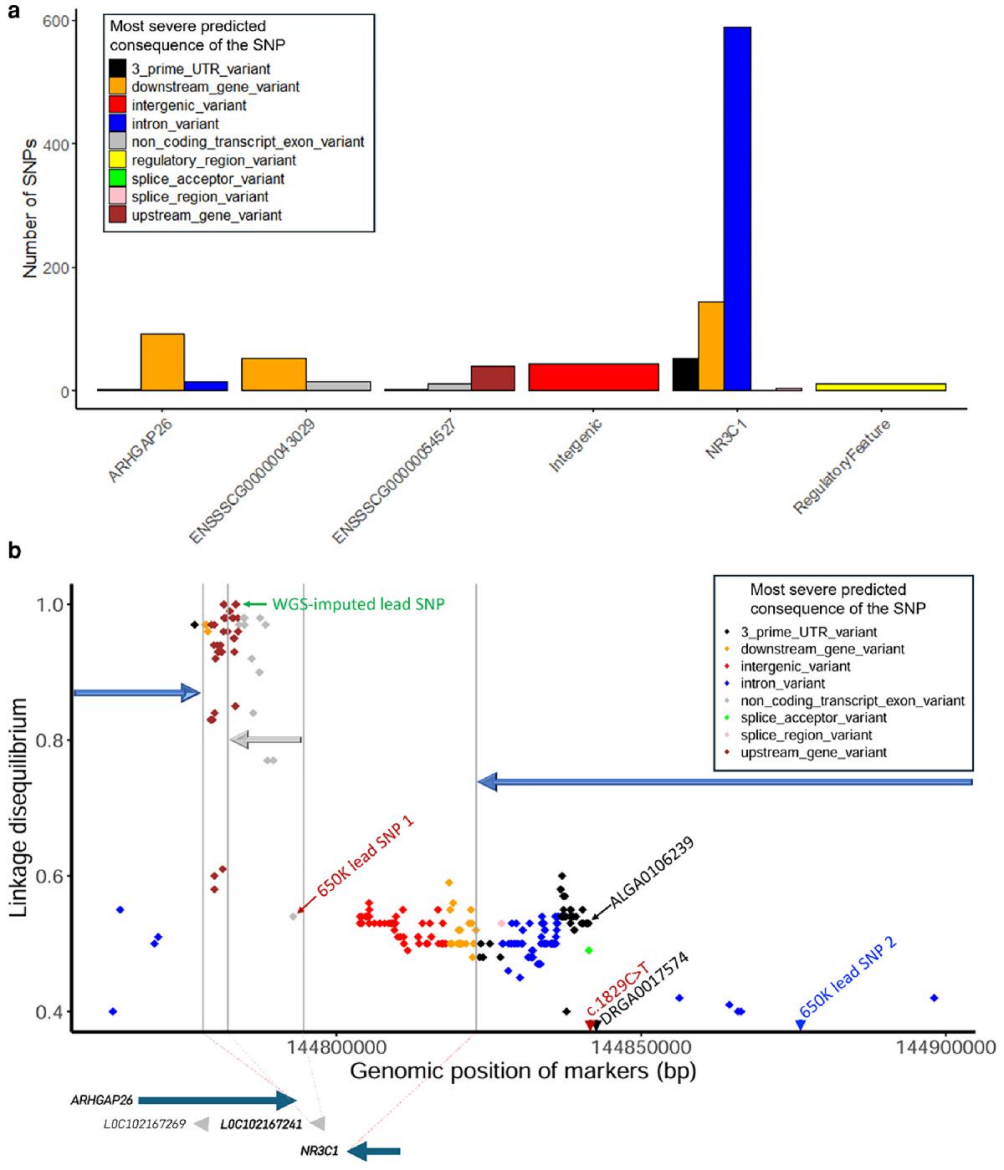
Predicted effects of SNPs in the QTL region for cortisol level that were in LD (*r*^2^ ≥ 0.4) with the WGS-imputed lead SNP on the genes in this region. a) The proportion of SNPs predicted to have different effects on the respective genes in this region. b) The most severe predicted effect of the SNPs that were in LD with the imputed lead SNP, clustered by their genomic positions with respect to the genes in the QTL region on SSC2 as predicted by the variant effect predictor tool of the Ensembl database. Also indicated are the WGS-imputed lead SNP (rs341258564), the 2 650 K lead SNPs (1 = rs336612332, 2 = rs330467924), the c.1829C > T causal variant for cortisol in blood identified by Muráni *et al*. (2012) (rs335303636), and 2 additional SNPs identified to be associated with cortisol in blood by Muráni *et al.,* (2012) and Terenina *et al*., (2025), i.e. ALGA0106239 (rs81333702) and DRGA0017574.

The WGS-imputed lead SNP in the major QTL neighbors the *GR* gene (*NR3C1*). However, we could not conclude that the imputed lead SNP is the causal variant for this QTL since many other SNPs are in high LD with this SNP (Supplementary Fig. 7d). The VEP of Ensemble (McLaren *et al*. 2016) predicted the WGS-imputed lead SNP to affect the lncRNAs in this region both as an upstream and as a downstream gene variant. Applying the VEP tool to the 272 SNPs that were in moderate to high LD (*r*^2^ ≥ 0.40) with the WGS-imputed lead SNP showed that they predicted to affect the *NR3C1* gene as “intron variants” (*n* = 117), as “downstream gene variants” (*n* = 83), or as “3′ UTR variants” (*n* = 42) (Fig. 5a). Supplementary Fig. 7e summarizes all the predicted consequences of these SNPs, while Fig. 5b shows their genomic position relative to the neighboring genes, including *NR3C1*, *ARHGAP26*, and 2 lncRNA genes (ENSSSCG00000054527 and ENSSSCG00000043029). Only 2 of the SNPs (rs345406620 and rs331270660) were predicted to affect splicing of the *NR3C1* gene (Fig. 5b). The SNPs that were in very high LD (*r*^2^ ≥ 0.8) with the WGS-imputed lead SNP mostly clustered in and around the lncRNA genes, with predicted effects on both the *ARHGAP26* and *NR3C1* genes. Overall, our fine-mapping approach narrowed the genomic region that includes the causal variant for the level of cortisol in hair of young and clinically healthy pigs but could not identify the causal variant due to high LD between SNPs in the region.

Muráni *et al.* (2012, 2016) previously reported and validated a gain-of-function mutation, c.1829C > T (rs335303636) in the *NR3C1* gene that affects cortisol levels in blood of pigs, using data from 4 commercial pig breeds (German Landrace, German Large White, Pietrain, and Duroc) and crossbred animals [Pietrain × (German Large White × German Landrace]. The position of this variant is annotated on Fig. 5b and did not coincide with any of the lead SNPs identified in the present study, i.e. the WGS-imputed lead SNP and the 2 650 K lead SNPs, which are also annotated on Fig. 5b. This causal variant reported by Muráni *et al.* (2012) is in exon 6 of the *NR3C1* gene and was shown to increase the sensitivity of the GR to glucocorticoids. The effect of this c.1829C > T substitution on cortisol levels in blood was also observed by Terenina *et al.* (2025), who evaluated cortisol levels 1 h after injecting 6-week-old Large White pigs with ACTH. This variant was not included in the 650 K SNP panel but was in our imputed WGS data. Of the 863 pigs with cortisol level data, 13.8% were heterozygous for this imputed causal variant, 86.2% were homozygous for the allele that increased blood cortisol levels in the study by Muráni *et al.* (2012), and no pigs were homozygous for the minor allele. To determine whether the Muráni *et al.* (2012) variant affected stress hormone levels in hair in our study, its WGS-imputed genotype was fitted as a fixed covariate in model (1). However, its minor allele substitution effect was essentially zero (−0.04 ± 0.04, *P* = 0.28) on the log scale. Additionally, fitting its genotype as a covariate in the GWAS model did not explain a substantial effect of the major QTL for cortisol (results not shown). Terenina *et al.* (2025), who also used the 650 K SNP panel for genotyping, also reported the 650 K lead SNP 1 in Fig. 5b that we identified to be associated with cortisol levels in blood of pigs after ACTH injection but not our 650 K lead SNP 2. Two other 650 K SNPs that were reported to be significantly associated with cortisol levels by both Muráni *et al.* (2012) and Terenina *et al.* (2025) were rs81333702 (denoted as ALGA0106239 by Muráni *et al.* 2012) and DRGA0017574 (this SNP was given an incorrect position of 14,484,166 bp instead of 144,842,462 bp in Table 3 of Terenina *et al*. 2025). Muráni *et al.* (2012) reported ALGA0106239 and DRGA0017574 to be in high LD (*r*^2^ = 0.78) with the causal mutation (c.1829C > T). However, in our population, SNP ALGA0106239 was not in LD with the imputed Muráni *et al*. (2012) causal mutation (*r*^2^ < 0.01) but it was in very high LD with our 650 K lead SNP 1 (*r*^2^ = 0.91, Fig. 4b).

Comparison of our results to those of Muráni *et al*. (2012) and Terenina *et al*. (2025), combined with the high LD in the region, breed or population differences, and possible errors in genotype imputation prevent us to determine whether QTL identified for cortisol level in hair of pigs in the present study is the same as that of cortisol levels in blood of pigs reported by Muráni *et al*. (2012) and Terenina *et al*. (2025). Further analyses are needed to genotype for the WGS-imputed lead SNP and for the causal variant reported by Muráni *et al.* (2012). Further studies should also consider resequencing this region for pigs with very high and very low hair cortisol levels or incorporate other molecular and gene editing strategies to identify or validate the causal mutation.

#### Effects of the major QTL for cortisol on gene expression

To assess the effect of the WGS-imputed lead SNP on the expression of candidate genes in the 5 Mb region centered around the QTL on SSC2 for cortisol, we used transcript abundance data of these genes in blood taken from 861 of the pigs used in this study and analyzed it using model (3). Results in Table 7 show no significant effects of an extra copy of the minor allele at the WGS-imputed lead SNP on transcript abundance of the candidate genes in blood of these pigs, including the GR gene. The same was true for the 2 lead SNPs based on the GWAS using the 650 K SNP panel, and for the causal variant that was reported by Muráni *et al*. (2012).

**Table 7.** Estimates of heritability (standard error, SE) and allele substitution effects of the significant SNPs and of the previously reported causal variant in the 1 Mb QTL for cortisol the transcript abundance of candidate genes in the blood transcriptome of young healthy pigs.

	No. of pigs	Heritability (SE)	Phenotypic variance (SE)	Allele substitution effect (SE) by SNP					
				rs81333702	rs331270660	rs336612332	rs330467924	rs335303636	rs341258564
*NR3C1*	861	0.05 (0.05)	0.88 (0.03)	0.01 (0.92)	−0.01 (0.80)	−0.01 (0.85)	−0.15 (0.12)	−0.04 (0.61)	0.02 (0.81)
*SRA1*	861	0.00	0.55 (0.03)	0.04 (0.45)	0.06 (0.30)	0.04 (0.44)	0.07 (0.43)	0.05 (0.09)	−0.02 (0.71)
*HDAC3*	861	0.03 (0.03)	0.49 (0.03)	−0.02 (0.76)	−0.02 (0.75)	0.01 (0.87)	0.08 (0.42)	0.05 (0.08)	−0.05 (0.41)
*ARHGAP26*	861	0.00	0.62 (0.03)	0.07 (0.24)	0.06 (0.26)	0.07 (0.23)	0.08 (0.40)	0.11 (0.21)	0.05 (0.42)

Results from the Farm Animal Genotype-Tissue Expression (FarmGTEx) project (Teng *et al*. 2024) reported the WGS-imputed lead SNP as an “enhancer QTL” for the *NR3C1* gene in testis and muscle but not in blood, suggesting tissue-specific effects of this major QTL on the expression of the *NR3C1* gene. Since the FarmGTEx project did not include expression data from hair tissues (follicles), we could not directly compare our findings to theirs. Interestingly, among SNPs that were in near-complete LD with the WGS-imputed lead SNP (i.e. ≥ 0.97), 4 were regulatory region variants according to VEP (including rs339385028, rs339251331, rs1109835066, and rs335962816), of which the latter is the WGS-imputed SNP that had PIP = 0.20 in the WGS GWAS described above. These results, combined with those of Teng *et al*. (2024), provide directional evidence that the regulatory region (most probably the enhancer region) of the *NR3C1* gene could harbor the causative mutation for cortisol levels in hair of young and clinically healthy pigs, although we could not validate this.

#### Bivariate GWAS to detect pleiotropic QTL

**Fig. 6.**
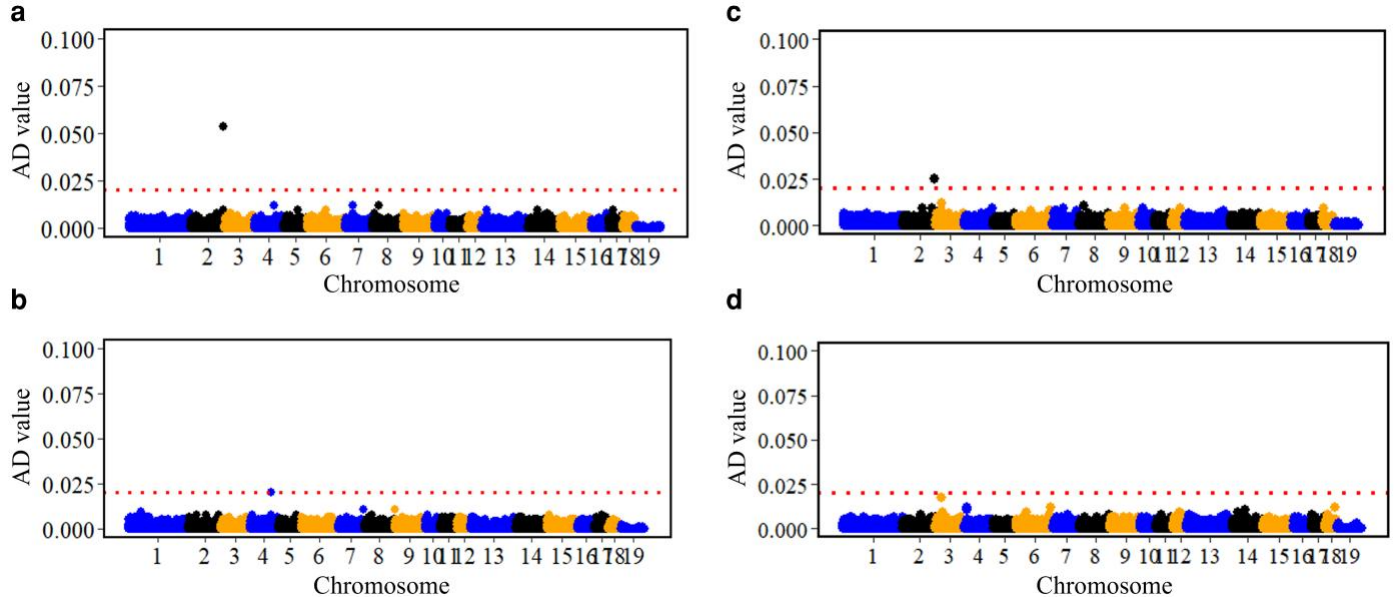
Bivariate GWAS results: Manhattan plots showing 0.25 Mb windows across the genome that were identified as pleiotropic QTL for a) cortisol and cortisone, b) cortisone and number of struggles, c) cortisol and number of vocalizations, and d) DHEA-S and number of vocalizations. The threshold line indicates the 2% AD threshold above which a window was considered be pleiotropic.

Genomic regions that drive pleiotropy between pairs of traits were identified using bivariate GWAS. Figure 6 shows Manhattan plots for pleiotropic QTL that were identified based on Ad values for 0.25 Mb windows. Supplementary Figs. 4–6, respectively, show similar Manhattan plots for pleiotropic regions among pairs of stress hormones, among pairs of backtest responses, and between pairs of stress hormones and backtest responses. A total of 3 pleiotropic QTL were identified. The major QTL for cortisol on SSC2 was pleiotropic for cortisol and cortisone, as well as for cortisol and VN. A pleiotropic QTL was also identified on SSC4 for cortisone and SN, while the QTL on SSC3 was pleiotropic for DHEA-S and VN. The few pleiotropic QTL identified indicate that there were no major regions that drive the covariance between the hormone levels and backtest responses but there could be many regions across the genome with small pleiotropic effects. These findings align with literature on the complex interplay of stress hormones during physiological homeostatic regulation of stress response (Tsigos *et al*. 2020), as well as with reports that behavioral traits in animals are highly polygenic (Santure *et al*. 2015; Laine and van Oers 2017; York 2018). Finally, combining bivariate GWAS results for cortisol and cortisone with the strong genetic correlation estimate between them (Table 4), we conclude that the QTL on SSC2 is a major genetic driver for levels of both glucocorticoids in hair of young and clinically healthy pigs.

### Gene set enrichment analyses

#### Enrichment based on univariate GWAS summary statistics

**Fig. 7.**
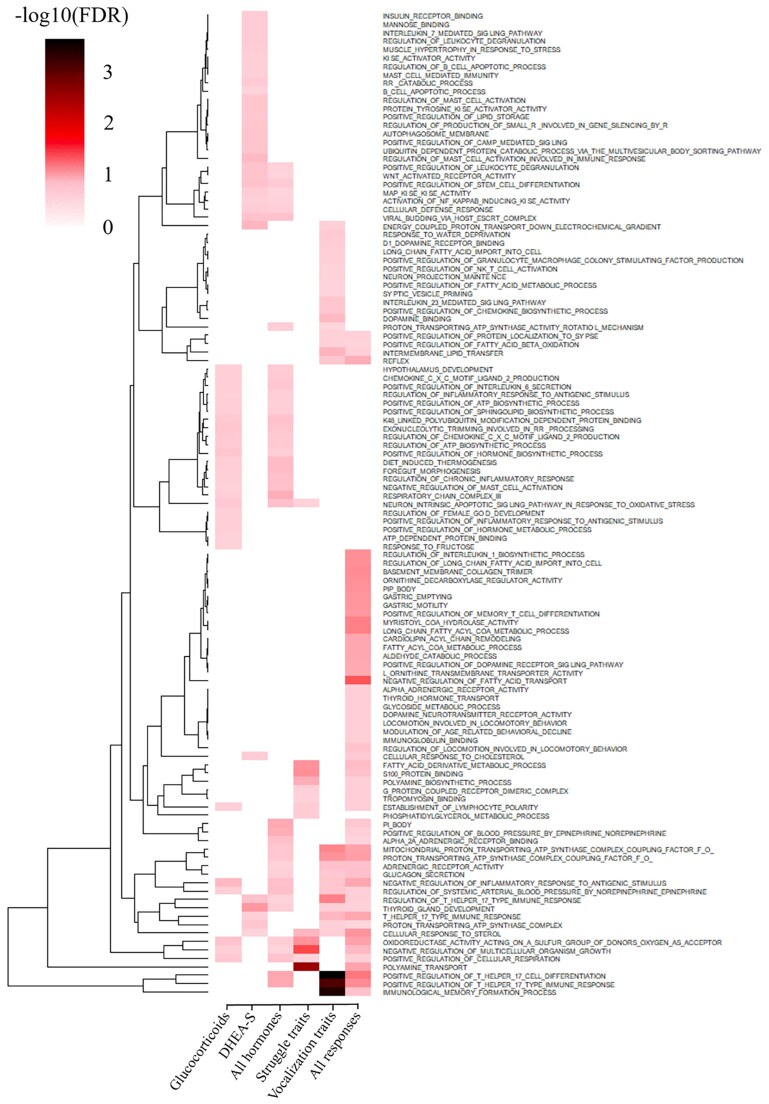
Heatmap showing GO terms that were significantly enriched (FDR ≤ 0.25) among windows beyond QTL identified in the univariate GWAS for different categories of stress hormones for glucocorticoids, DHEA-S, and all hormones combined, and of different categories of backtest responses: struggles, vocalizations, and all responses combined. The -log10(FDR) was used to quantify the level of enrichment.

Figure 7 shows GO terms that were significantly enriched (FDR ≤ 0.25) among 0.25-Mb windows beyond the identified QTL for different groups of the evaluated stress response. Windows that were associated with more variance for glucocorticoids were enriched for BPs related to production of energy, such as ATP biosynthesis, diet metabolism, gut morphogenesis, and enhanced cellular respiration. These are attributes of the catabolic nature of glucocorticoids aimed at availing energy for the body stay on high alert under stressful conditions (Thau *et al*. 2023). These windows were also enriched for biological processes related to regulating the production of chemokines and negatively regulating inflammatory response to antigenic stimulus and mast cell activation, which are part of the anti-inflammatory characteristics of glucocorticoids. Kadmiel and Cidlowski (2013) also reported that glucocorticoids induced apoptosis of proinflammatory T cells, suppress B cell antibody production, and reduce neutrophil migration during inflammation.

Windows associated with DHEA-S were enriched for biological processes related to enhancing immune response such as activating mast cells, regulating T-helper 17 immune response, B cell activity, and enhancing leukocyte degranulation (Fig. 7). Leukocyte degranulation is a mechanism by which white blood cells combat infection, inflammation, and other immune system challenges (Felix et al. 2018). They were also enriched for terms related to regulating transcription and silencing of genes through miRNA, ubiquitin-dependent protein catabolism, and activation of cell-signaling protein kinase pathways, such as that of the transcription factor NF-κB, serine-threonine kinase, tyrosine kinase, and the mitogen-associated protein kinase (Fig. 7).

When GWAS results for all backtest responses were combined, windows associated with higher variance were enriched for features related to regulation of the neuroendocrine signaling pathways involving dopamine, epinephrine, and norepinephrine, and their receptors (e.g. the alpha-1A adrenergic and alpha-1B adrenergic receptors), as well as for positive regulation of blood pressure in response to these neuroendocrine signals (Fig. 7). These windows were also enriched for features involved in the production of metabolic energy (e.g. gastric motility and emptying, regulated fatty acid metabolism, and glucagon secretion) and regulation of locomotion behavior. Such processes are of biological relevance during physical response to stressors in animals, since the body requires energy mobilized from cellular respiration using stored fatty acids and glucose retrieved from glycogen stores and dietary sources. These results support existing literature showing that physical stress response is generally modulated through coordinated activation and control of neuroendocrine signaling to mobilize and reallocate resources based on the modality and intensity of the stressor, as perceived by the animal (Ulrich-Lai and Herman 2009; Etim *et al*. 2013).

Windows associated with vocalization traits (VN and VI) were strongly enriched for features related to immune response, such as activation of T-helper cell differentiation and response, and immunological memory, among others. The link between vocalizing and the immune system in pigs during stress response is not clear. Geverink *et al*. (2004) found no significant difference in immune response of high and low responding pigs to a DNP-KLH immunization, while Bolhuis *et al*. (2004)found that KLH-specific lymphocyte proliferation was higher in high- than low-resisting pigs but also registered significant interactions of housing with the behavioral response of pigs to the backtest. However, vocalization has been shown to aid identification of the most frequent stressful conditions at farrowing, such as pain, cold, and hunger (da Silva Cordeiro *et al*. 2013). Furthermore, Jouda *et al*. (2019)found that stimulation of the immune system affected the ultrasonic calling of pups in mice and rats. These results make this area worth investigating further.

Windows associated with struggle traits (SI and SN) were strongly enriched for polyamine biosynthesis and transport, and for cellular respiration (Fig. 7). Transport of polyamine is mediated by solute carrier (SLC) and ATP-binding cassette (ABC) transporters (Sagar *et al*. 2021) and they are involved in protein synthesis, mitochondrial function, and energy production (Bekebrede *et al*. 2020), which is required when pigs struggle. Interestingly, a member of the SLC family (*SLC16A3*) was a candidate gene for a QTL identified for VI, while a member of the ABC transporters (*ABCB5*) was a candidate gene for the QTL identified for VN (Table 5). Given the strong genetic correlation estimates among the backtest responses (Table 4), our results suggest that polyamine transport is relevant during physical/behavioral response to noninfectious stressors. On the other hand, windows associated with SI and SN were moderately enriched (FDR < 0.15) for S100 binding proteins and for tropomyosin binding (FDR ≤ 0.25) (Fig. 7), which reinforces the importance of the suggested region on SSC4 for these 2 traits (Table 5), as it harbors the *TMOD4* and many S100 protein genes. Members of the S100 protein family are known to participate in storage and transport of calcium (Singh and Ali 2022), a key component of muscle contraction. They are also involved in regulating energy metabolism (Sreejit et al. 2020), thereby possibly influencing the struggle traits, in addition to being involved in many inflammatory pathways, which connects coping style to immune response. Tropomodulin 4 regulates filament elongation and depolarization along with tropomyosin in muscle (Almenar-Queralt *et al*. 1999), which are required when pigs struggle during the backtest.

Overall, the GSEA of genomic regions, other than the declared QTL, that were associated with a trait or group of traits revealed more information on the BPs that may be modulating response to noninfectious stressors in pigs, with effects distributed across the genome in regions with smaller effects. This approach, for example, reproduced the opposing roles of glucocorticoids and DHEA-S on immune-related mechanisms during stress response, such as those on mast cell activation and others, which aligns with reports by McNelis *et al*. (2013) and Whitham *et al*. (2020). Finally, these enrichment results support available literature that response to noninfectious stressors, as reflected by stress hormone levels in hair, involves the immune system, neuroendocrine signaling, cellular signaling, and body energy metabolism. Therefore, analyzing the biological relevance of these regions using GSEA complemented the significant GWAS findings in understanding the underlying genetic and biological basis of stress response in pigs.

#### Enrichment based on bivariate GWAS

**Fig. 8.**
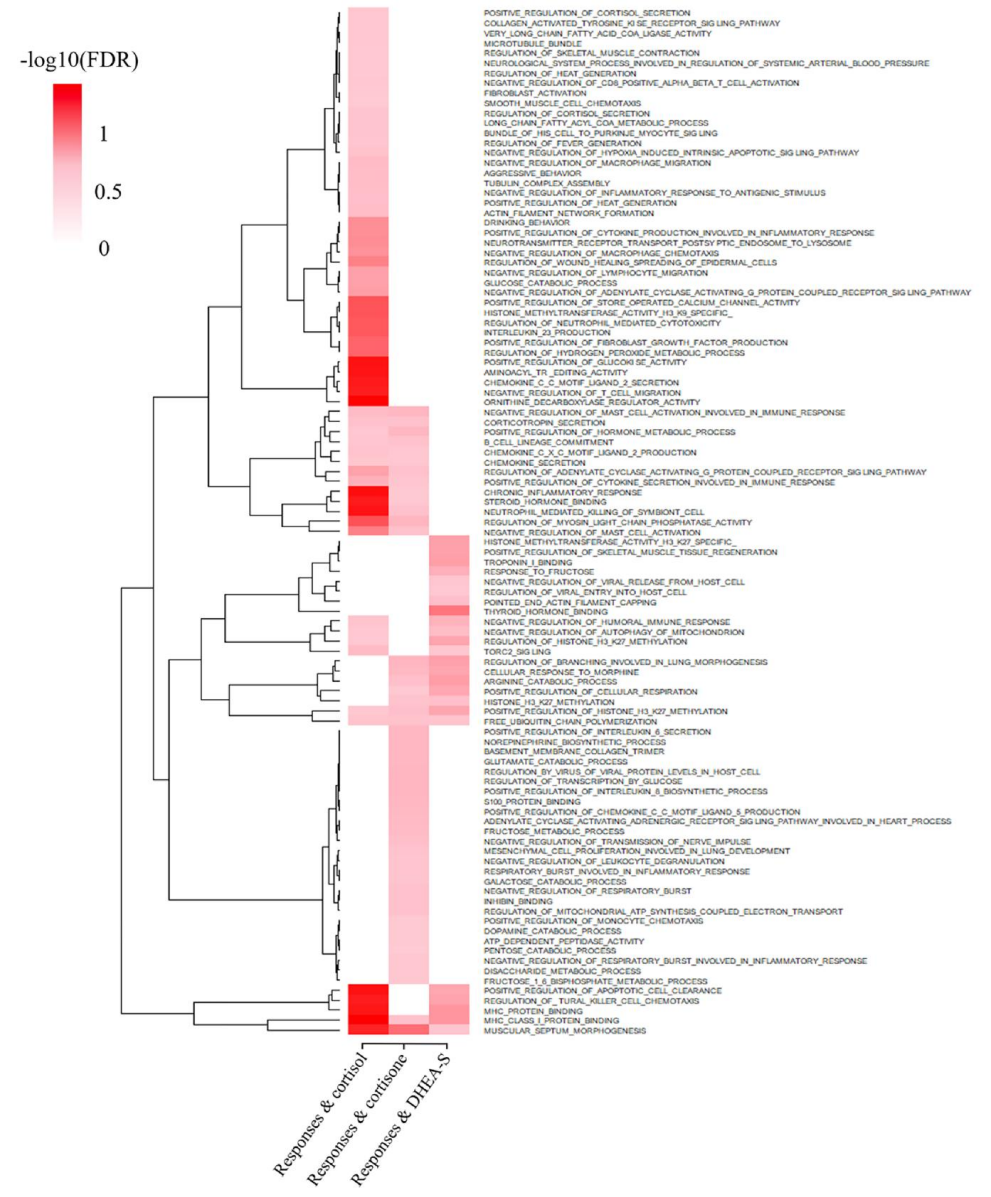
Heatmap showing GO terms that were significantly enriched (FDR ≤ 0.25) among windows that did not reach significance in bivariate GWAS for stress hormones and the backtest responses.

Enrichment results for regions associated with pleiotropy between pairs of traits are shown in Fig. 8. Pleiotropic regions for cortisol and backtest responses were strongly enriched (FDR ≤ 0.25) for features related to negative regulation of immune-related mechanisms (macrophage chemotaxis, mast cell activation, and lymphocyte migration), increased lipid metabolism, increased glucose metabolism, and enhanced muscle and skeletal movements, with associated heat generation. These windows were also enriched for features related to aggressive behavior, which aligns with the moderate positive genetic correlation observed between cortisol and all backtest responses.

Pleiotropic windows for cortisol with backtest responses and for cortisone with backtest responses were enriched for biological processes related to steroid hormone binding, cytokine production, and negative regulation of mast cell activation (Fig. 8). Interestingly, features related to the respiratory burst, which is the principal effector mechanism used to kill internalized pathogens following phagocytosis by macrophages and neutrophils (Iles and Forman 2002; Desforges *et al*. 2016), were enriched among windows that were pleiotropic for backtest responses with cortisone, but not with cortisol. Pleiotropic windows for DHEA-S with backtest responses were enriched for features involved in thyroid hormone activity and regulating susceptibility of cells to viral infection (Fig. 8).

Generally, pleiotropic windows for all pairs of stress hormone and backtest responses were significantly enriched (FDR ≤ 0.25) for biological features related to major histocompatibility complex (MHC) binding, as well as those related to regulating gene transcription by ubiquitin chain polymerization and epigenetic methylation of histone H3K27. This suggests the relevance of the MHC region to an animal's response to not only infectious stressors, as reported by Cheng *et al.* (2022) for the NDCM, but also to noninfectious stress such as handling, transportation, weaning, and others.

## Conclusion

This study is the first to report the genetic basis of stress hormone levels in hair of young and clinically healthy pigs. Results suggest that the level of cortisol in hair of young and clinically healthy pigs that are responding to noninfectious stressors is a potential genetic indicator of their coping response style. Based on heritability estimates of these retroactive measures of stress response, these results highlight the potential of using stress hormone traits from hair to select for pigs that cope better with noninfectious stress. Subsequent work will evaluate the genetic basis of stress hormone traits in hair grown during response to infectious stress, which, combined with the current results, will facilitate selecting pigs for general stress resilience. GWAS results identified a major QTL for cortisol levels in hair of these pigs on SSC2 and fine mapping established that the lead SNP for this QTL neighbors the glucocorticoid receptor gene (*NR3C1*). Fine mapping this region revealed that the causal variant for this major QTL may be around the enhancer region of the *NR3C1* gene but some LD patterns in the region connect it to a causal mutation that has been reported earlier for cortisol levels in blood of pigs. However, because of the high LD in the region, breed or population differences, and possible errors in genotype imputation, we could not identify the actual causal variant for this major QTL nor confirm whether it is the same as that reported previously for cortisol levels in blood of pigs. However, we point to a region that includes the *ARHGAP26* and *NR3C1* genes as the most prominent location of the causal variant. Other QTL with relatively minor effects were reported for DHEA-S, cortisol/DHEA-S, and cortisone/DHEA-S, as well as the first GWAS results for backtest responses, including QTL for VN and VI.

Results show the similarity in the genetic control of levels of cortisol and cortisone in hair but failed to establish this relationship for DHEA and DHEA-S because DHEA in hair of these pigs was not heritable. In addition, this study highlights the complexity in the genetic modulation of the physiological and physical responses to noninfectious stressors in pigs, showing that response to noninfectious stress is highly polygenic, with numerous regions across the genome explaining small proportions of the genetic (co)variance. Functional enrichment analyses, however, showed that genomic regions associated with stress hormone levels in hair beyond the identified QTL were enriched for biological processes that provide more information on the biology of response to noninfectious stressors and of the coping style of response of pigs to these stressors and included biological processes associated with immune response. Subsequent studies will evaluate responses to infectious stressors of these same pigs, as part of the NDCM, including stress hormone levels in hair grown during exposure to infectious stressors, as well as associated disease resilience traits in terms of performance and clinical disease traits.

## Supplementary Material

iyaf092_Supplementary_Data

## Data Availability

The data analyzed in this study were collected on animals that were provided by and are part of the commercial breeding programs of the 7 investing member businesses of PigGen Canada (https://piggencanada.org/, last accessed 10/30/2024). The data and samples generated on these animals are thus confidential and protected as intellectual property or as trade secrets. As a result, the data analyzed in this study are not publicly available but are stored in a secure database at the University of Alberta. Data can, however, be made available on reasonable request, as detailed in supplementary data access procedure file. Supplemental material available at GENETICS online.
