# Supplementary material for: Genetic analysis of stress hormone levels in hair of healthy nursery pigs and their relationships with backtest responses
Source: Genetics. 2025 May 14;230(4):iyaf092. doi: 10.1093/genetics/iyaf092 (PMC12341881; doi:10.1093/genetics/iyaf092)
Supplement: iyaf092_Supplementary_Data [file iyaf092_supplementary_data.zip › Supplemental_Figure_Legends_GENETICS-2025-308121.docx]

**Supplemental Figure Legends**

**Figure S1.** Plots showing the relationship between the levels of stress hormones extracted from hair samples and the length of time samples were stored between grinding of hair and extraction of stress hormones. The effect of the storage length was used as a fixed covariate in the univariate GWAS models for all hormones and their ratios for consistency.

**Figure S2.** Manhattan plots showing the percentage of genetic variance explained by non-overlapping 1 Mb windows for the levels of cortisone, DHEA, and cortisol/cortisone. A QTL was any window that explained more than 1% of the estimated genetic variance (above the red line).

**Figure S3.** Manhattan plots showing the percentage of genetic variance explained by non-overlapping 1 Mb windows for responses to the backtest: a) Vocalization number; b) Vocalization intensity; c) Struggles number; d) Struggle intensity. A QTL was defined as a 1 Mb window more than the 1% of the estimated genetic variance (above the red line).

**Figure S4.** Manhattan plots showing non-overlapping 0.25 Mb windows associated with the pleiotropic effects for levels of cortisol and DHEA-S, and for levels of cortisone and DHEA-S in hair of young healthy pigs. The red line indicates a 2% absolute difference threshold above which a window was considered a pleiotropic QTL for the pair of hormones.

**Figure S5.** Manhattan plots showing non-overlapping 0.25 Mb windows associated with the pleiotropic effects for pairs of backtest responses: a) struggles number and struggle intensity, b) vocalization intensity and struggle intensity, c) vocalization intensity and struggles number, d) vocalization number and struggle intensity, e) vocalization number and struggles number, f) vocalization number and vocalization intensity. The red line indicates a 2% absolute difference threshold above which a window was considered a pleiotropic QTL for the pair of responses

**Figure S6.** Manhattan plots showing non-overlapping 0.25 Mb windows associated with the pleiotropic effects for pairs of stress hormones and backtest responses: a) cortisol and backtest responses, b) cortisone and backtest responses, c) DHEA-S and backtest responses. The red line indicates a 2% absolute difference threshold above which a window was considered a pleiotropic QTL for the pair of traits.

**Figure S7.** a) Manhattan plot showing the percentage of genetic variance in hair cortisol that was explained by the QTL on SSC2 when genotypes of the marker in the 1 Mb QTL region were imputed to whole genome sequence, and the plot of the posterior inclusion probability showing the imputed markers with the posterior inclusion probability ≥ 1%; b) the heatmap showing the LD (r^2^) between the significant imputed markers (green) with those that were most frequently sampled when using the 650K panel (red and blue), and with the imputed SNP that had earlier been reported to be the causal variant for cortisol in blood plasma (black-bold); c) Manhattan plot showing the percentage of genetic variance that was explained by the QTL on SSC2 when the effect of the imputed lead SNP (rs341258564) was fitted as a fixed covariate in the univariate GWAS model d) the LD between other markers in the QTL region with the significant imputed SNP (at position 0); e) a pie chart showing the frequency of the predicted effects of the imputed markers in LD (r^2^ ≥ 0.4) with the imputed lead SNP in the QTL.
