# Supplementary figures and images for: Genetic analysis of stress hormone levels in hair of healthy nursery pigs and their relationships with backtest responses
Source: Genetics. 2025 May 14;230(4):iyaf092. doi: 10.1093/genetics/iyaf092 (PMC12341881; doi:10.1093/genetics/iyaf092)

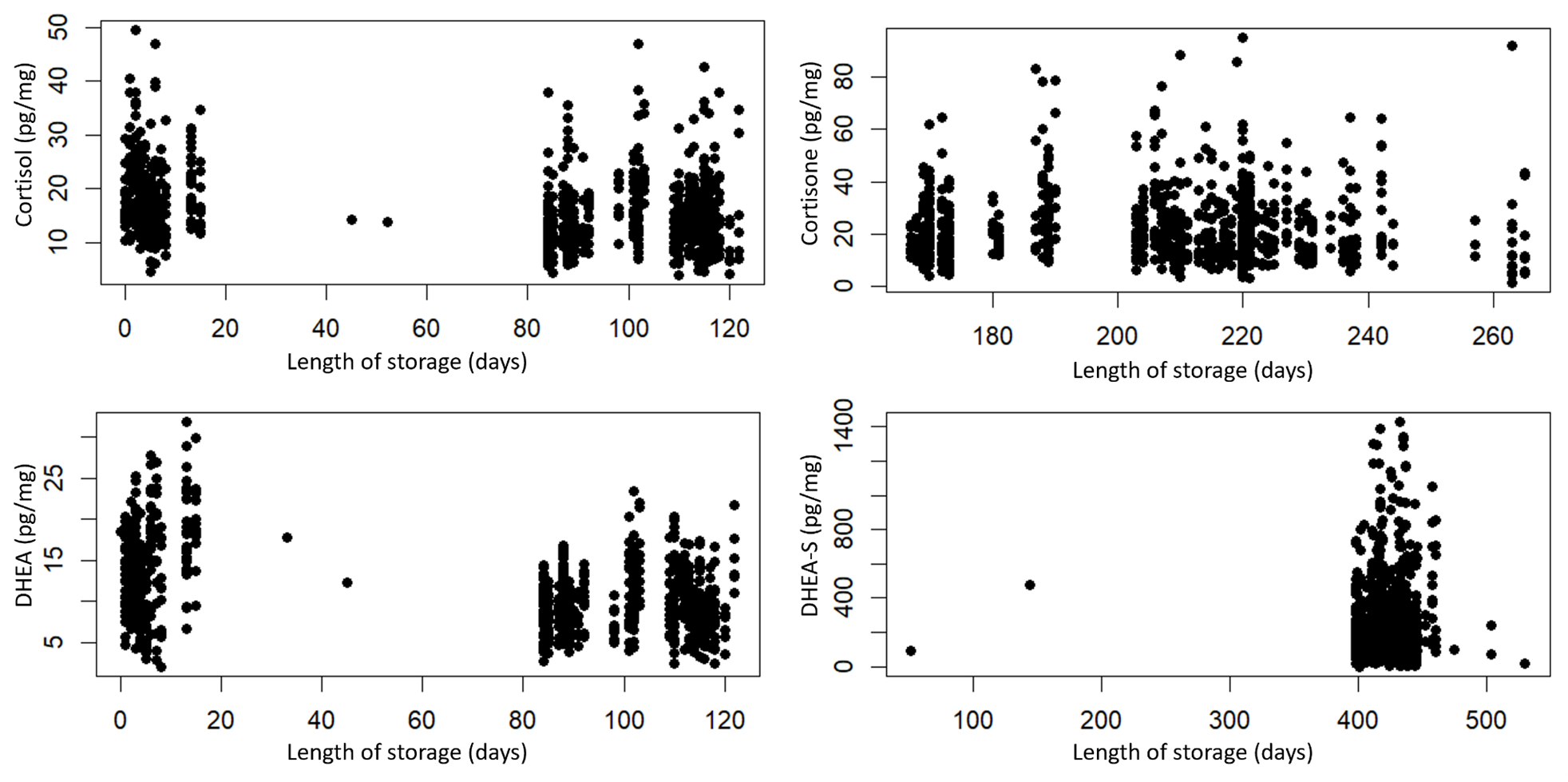

Supplement: iyaf092_Supplementary_Data [file iyaf092_supplementary_data.zip › Figure_S1_GENETICS-2025-308121.png]

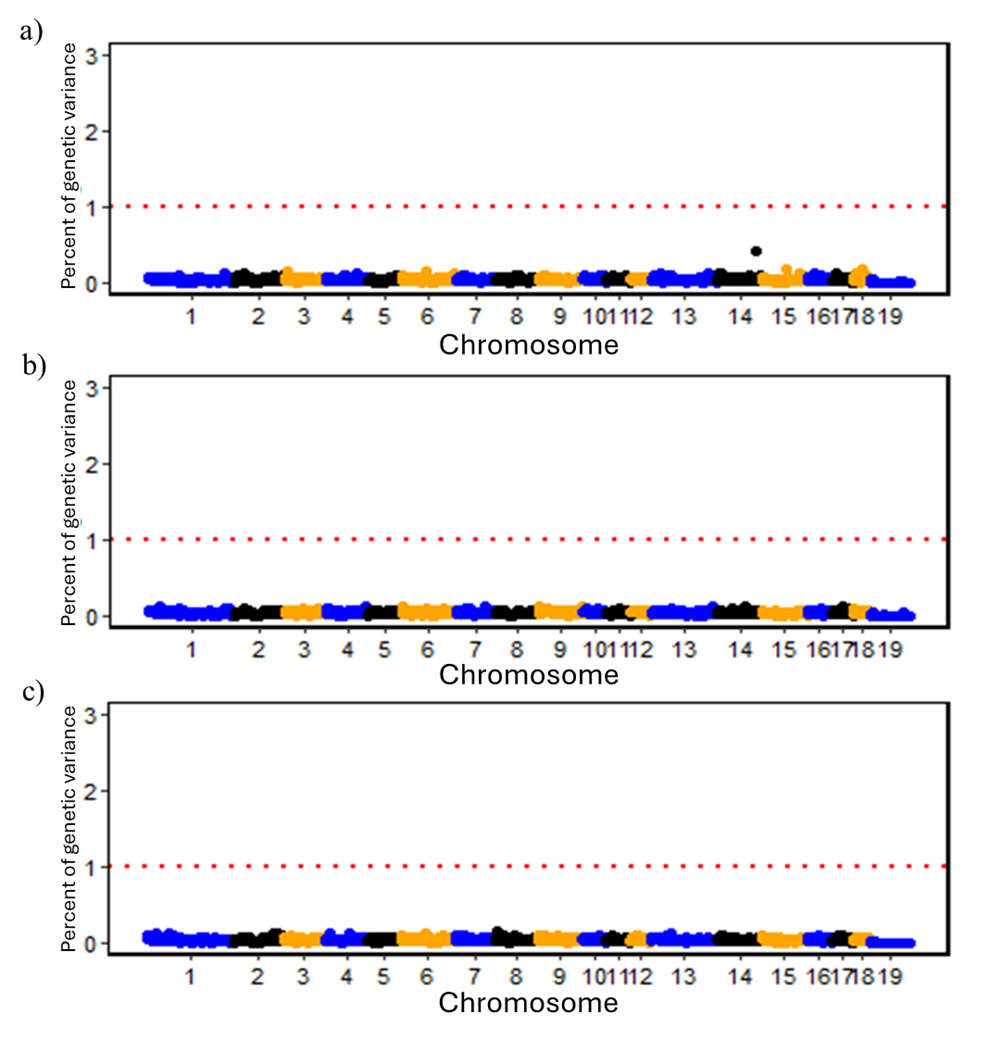

Supplement: iyaf092_Supplementary_Data [file iyaf092_supplementary_data.zip › Figure_S2_GENETICS-2025-308121.png]

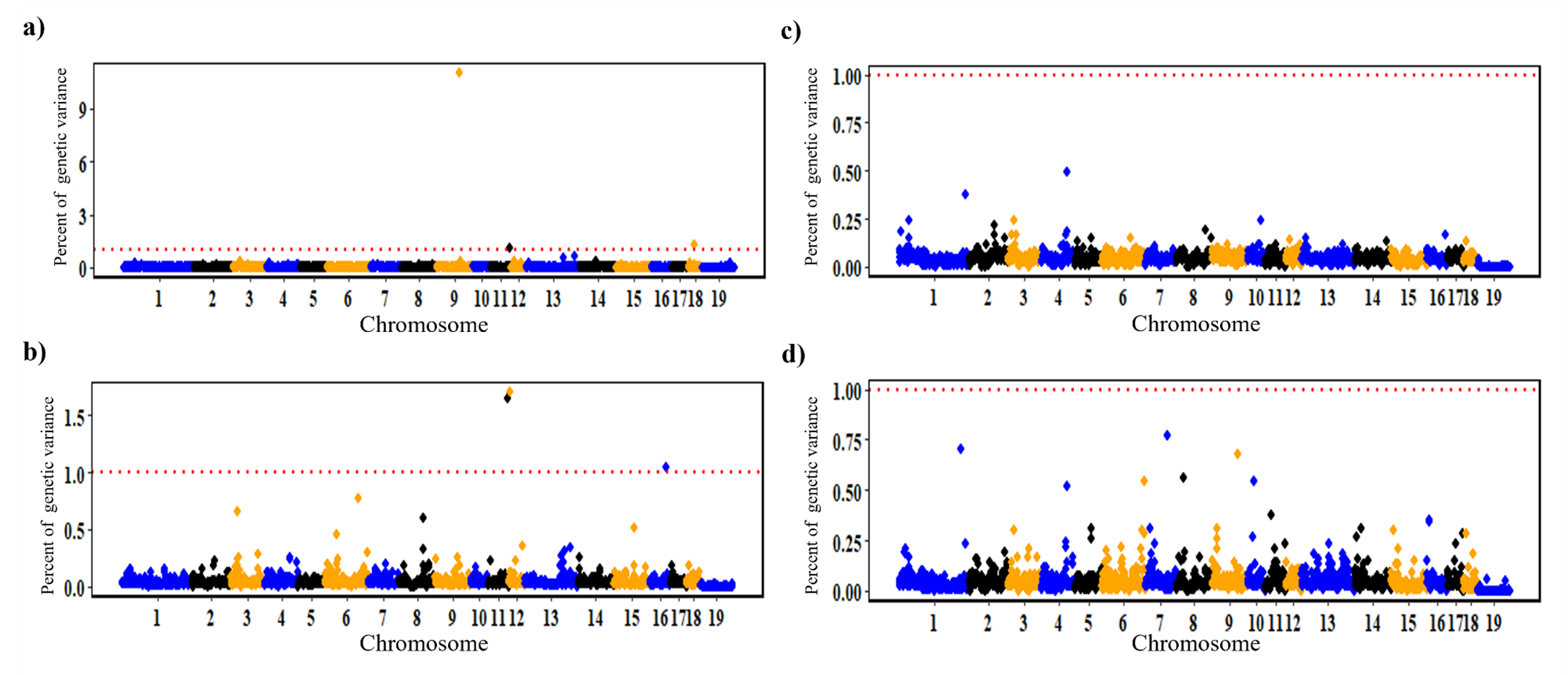

Supplement: iyaf092_Supplementary_Data [file iyaf092_supplementary_data.zip › Figure_S3_GENETICS-2025-308121.png]

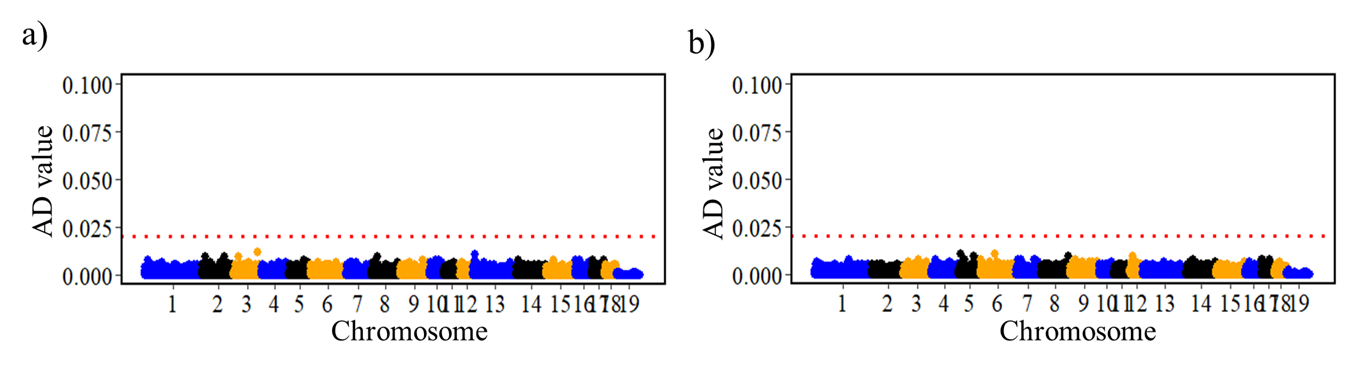

Supplement: iyaf092_Supplementary_Data [file iyaf092_supplementary_data.zip › Figure_S4_GENETICS-2025-308121.png]

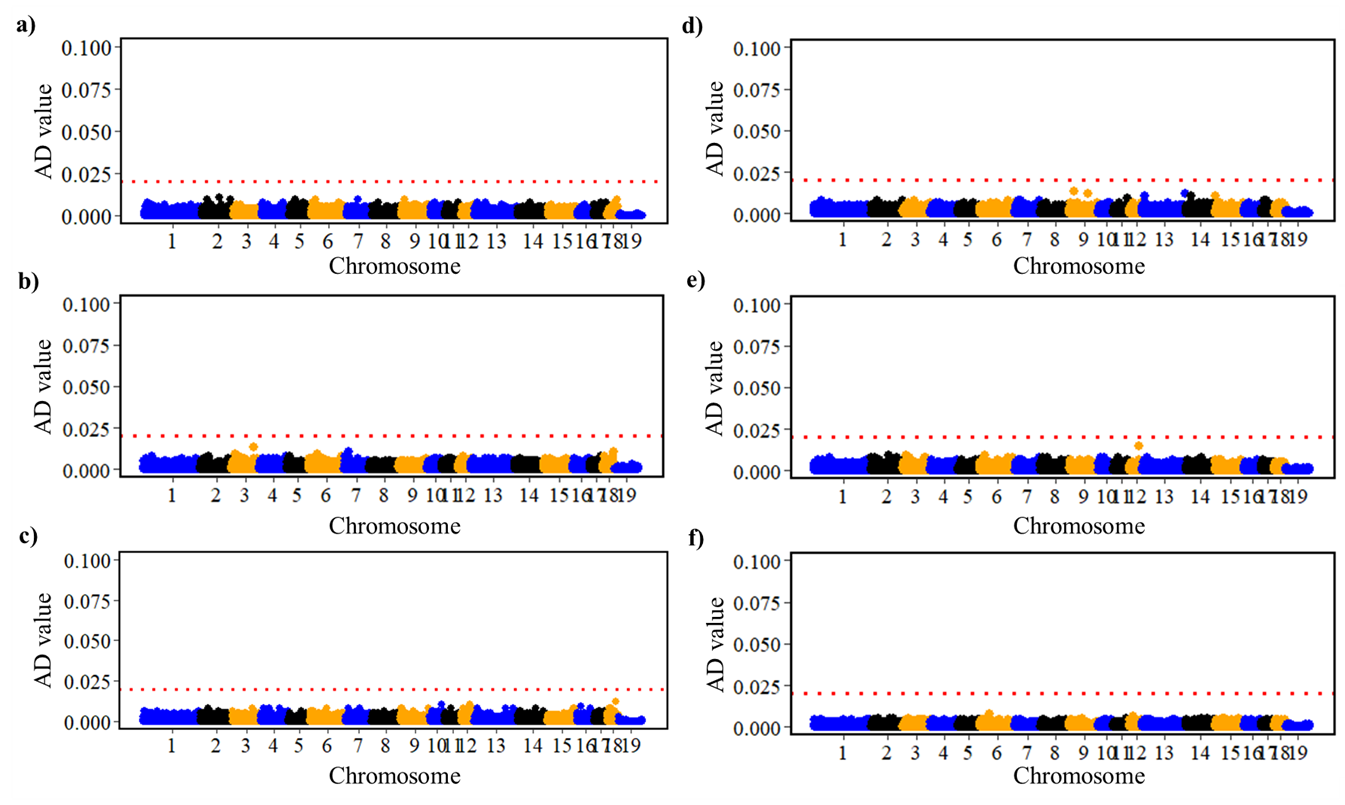

Supplement: iyaf092_Supplementary_Data [file iyaf092_supplementary_data.zip › Figure_S5_GENETICS-2025-308121.png]

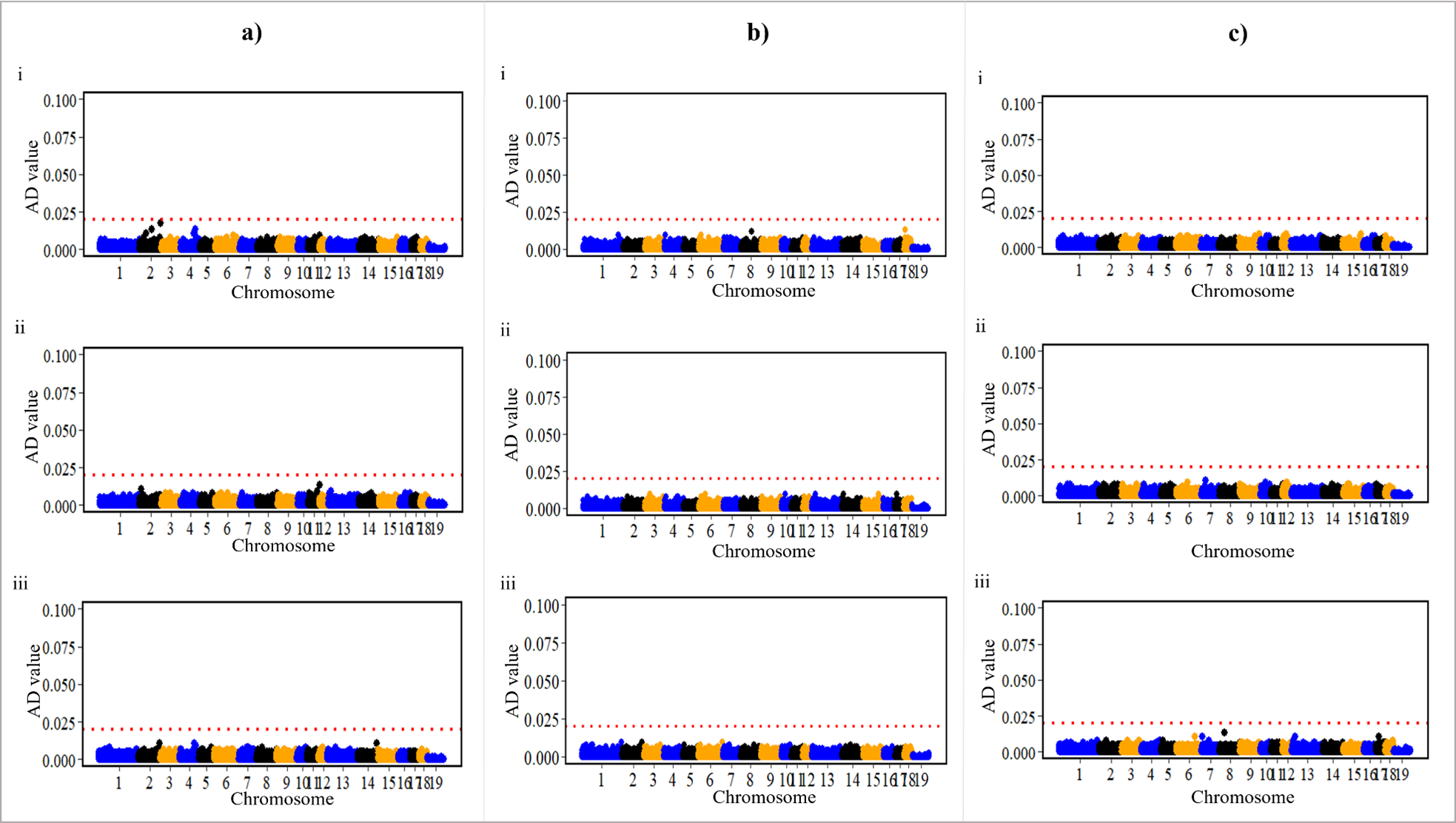

Supplement: iyaf092_Supplementary_Data [file iyaf092_supplementary_data.zip › Figure_S6_GENETICS-2025-308121.png]

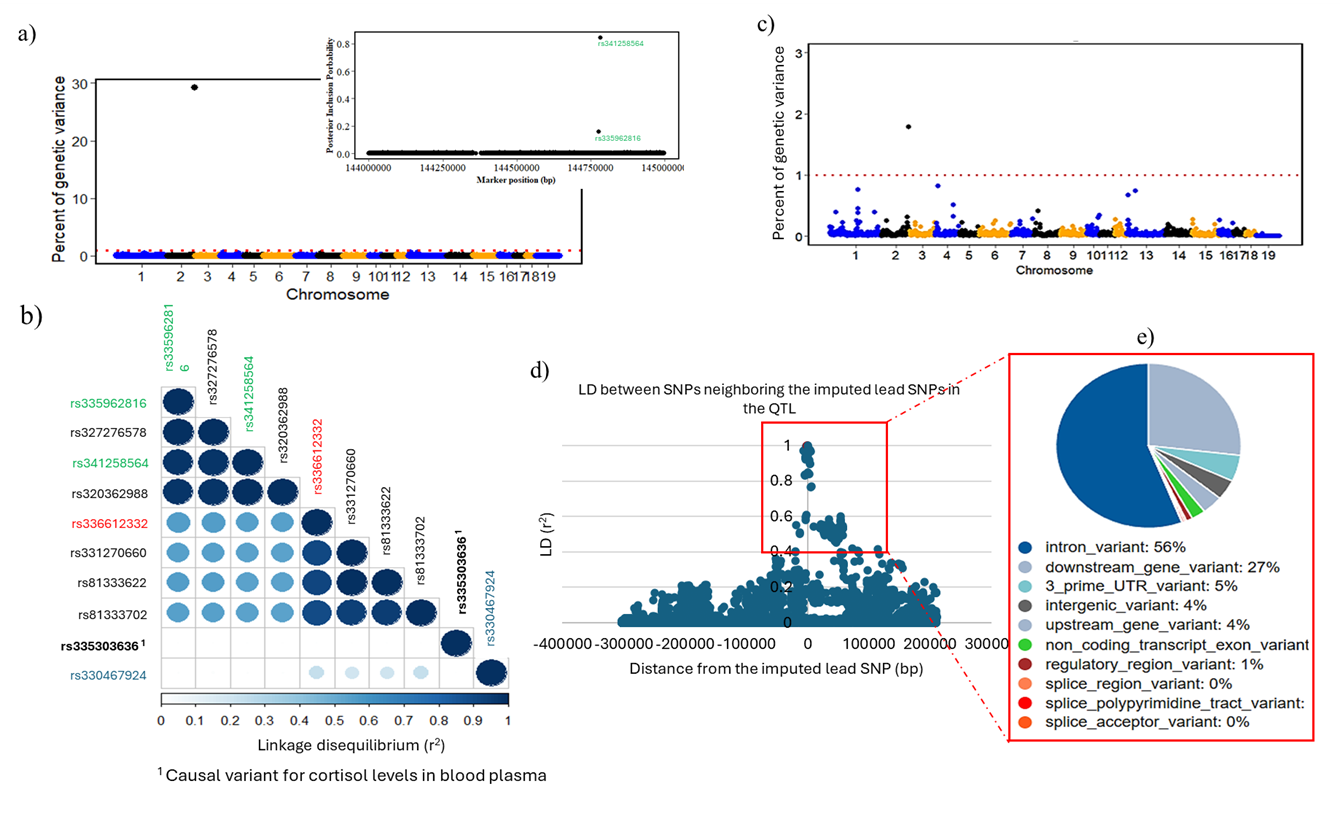

Supplement: iyaf092_Supplementary_Data [file iyaf092_supplementary_data.zip › Figure_S7_GENETICS-2025-308121.png]
